# Therapeutically expanded human regulatory T-cells are super-suppressive due to HIF1A induced expression of CD73

**DOI:** 10.1038/s42003-021-02721-x

**Published:** 2021-10-14

**Authors:** Lorna B. Jarvis, Daniel B. Rainbow, Valerie Coppard, Sarah K. Howlett, Zoya Georgieva, Jessica L. Davies, Harpreet Kaur Mullay, Joanna Hester, Tom Ashmore, Aletta Van Den Bosch, James T. Grist, Alasdair J. Coles, Hani S. Mousa, Stefano Pluchino, Krishnaa T. Mahbubani, Julian L. Griffin, Kourosh Saeb-Parsy, Fadi Issa, Luca Peruzzotti-Jametti, Linda S. Wicker, Joanne L. Jones

**Affiliations:** 1 Department of Clinical Neurosciences, University of Cambridge, Cambridge, UK; 2 JDRF/Wellcome Diabetes and Inflammation Laboratory, Wellcome Centre for Human Genetics, Nuffield Department of Medicine, NIHR Oxford Biomedical Research Centre, University of Oxford, Oxford, UK; 3 Department of Medicine, University of Cambridge, Cambridge, UK; 4 Department of Nuffield Department of Surgical Sciences, University of Oxford, Oxford, UK; 5 Department of Biochemistry and Cambridge Systems Biology Centre, University of Cambridge, Cambridge, UK; 6 Department of Radiology, University of Cambridge, Cambridge, UK; 7 Department of Surgery, University of Cambridge, Cambridge, UK; 8 Imperial College London Dementia Research Institute & Section of Biomolecular Medicine, Department of Metabolism, Digestion and Reproduction, Imperial College London, London, UK

**Keywords:** Graft-versus-host disease, Autoimmune diseases, Regulatory T cells, Immunosuppression

## Abstract

The adoptive transfer of regulatory T-cells (Tregs) is a promising therapeutic approach in transplantation and autoimmunity. However, because large cell numbers are needed to achieve a therapeutic effect, in vitro expansion is required. By comparing their function, phenotype and transcriptomic profile against ex vivo Tregs, we demonstrate that expanded human Tregs switch their metabolism to aerobic glycolysis and show enhanced suppressive function through hypoxia-inducible factor 1-alpha (HIF1A) driven acquisition of CD73 expression. In conjunction with CD39, CD73 expression enables expanded Tregs to convert ATP to immunosuppressive adenosine. We conclude that for maximum therapeutic benefit, Treg expansion protocols should be optimised for CD39/CD73 co-expression.

## Abstract (web summary)

Jarvis et al demonstrate that expanded human Tregs switch their metabolism to aerobic glycolysis and show enhanced suppressive function through hypoxia-inducible factor 1-alpha (HIF1A)-driven acquisition of CD73 expression, which along with CD39, enables expanded Tregs to convert ATP to immunosuppressive adenosine. Given this, the data suggests that Treg expansion protocols should be optimised for CD39/CD73 co-expression to enhance therapeutic potential.

## Introduction

Regulatory T cells (Tregs) are a subset of CD4^+^ T cells that play an indispensable role in maintaining self-tolerance and in controlling potentially harmful excessive immune responses to foreign antigens. They are characterized by surface expression of the high affinity IL-2 α-chain receptor (CD25), low expression of the IL-7 α-chain receptor (CD127) and by demethylation at a conserved region within intron 1 of the *FOXP3* gene called the Treg-specific demethylated region (TSDR), leading to constitutive expression of their master transcription factor FOXP3.

Multiple mechanisms have been proposed for Treg suppressive function, including: (i) consumption of IL-2; (ii) production of inhibitory cytokines; (iii) inhibition of antigen presenting cell maturation and function via the interaction of CTLA4 with CD80/86 on dendritic cells and (iv) the conversion of proinflammatory extracellular ATP to immunoregulatory adenosine through the action of the surface ectonucleotidases CD39 (ectonucleoside triphosphate diphosphohydrolase-1) and CD73 (ecto-5’-Nucleotidase). Mechanistically, CD39 degrades ATP into AMP, which is then hydrolyzed by CD73 into adenosine (ADO). The importance of ADO mediated suppression for human Tregs is unclear, as unlike murine Tregs it is generally accepted that they do not express CD73^1^.

Given the critical role Tregs play in controlling immune responses, there is a great deal of interest in their therapeutic potential, particularly in the treatment of autoimmune diseases and in treating, or preventing, complications from transplantation, such as graft-versus-host disease (GvHD) following hematopoietic stem cell transplantation (HSCT), or graft rejection following solid organ transplantation. Numerous proof-of-principal pre-clinical studies have demonstrated their efficacy^2–5^, several phase I/II studies have demonstrated their safety and tolerability^6^, and numerous clinical trials are underway (clinicaltrials.gov and reviewed^7^). However, despite their potential, Treg therapy poses a number of significant challenges, not least of which is cell number. Tregs make up only 5% of circulating CD4 T cells, therefore production of therapeutic doses capable of altering immune responses requires intensive ex vivo expansion. Most protocols involve broad T-cell receptor (TCR) stimulation of magnetically separated or flow-sorted Tregs together with provision of IL-2 and the mTOR inhibitor rapamycin for periods of up to 36 days^8^. Rapamycin is an immunosuppressant that forms a gain-of-function complex with the FK506-binding protein (FKBP12), which then binds to and inhibits mTOR complex 1 (mTORC1), inhibiting T-cell proliferation. At low doses, rapamycin preferentially inhibits T effector (Teff) proliferation, preventing their outgrowth, so improving Treg purity^9^.

We and others have previously shown that expanded Tregs (expTregs) have increased suppressive function when compared to their ex vivo activated counterparts^10–12^ but it is unclear why, and currently there is no consensus on which phenotypic characteristics best predict therapeutic efficacy. Here we show that acquisition of CD73, leading to increased CD39/CD73 co-expression and subsequent ability to produce immunosuppressive adenosine from ATP, best explains the increased suppressive capacity of human expTregs. Furthermore, we demonstrate that stable CD73 expression occurs as expTregs switch their metabolism from oxidative phosphorylation (OXPHOS) to aerobic glycolysis, and that CD73 expression is driven by hypoxia-inducible factor 1-alpha (HIF1A). Given the clinical importance of optimizing their suppressive function, we suggest that therapeutic Treg expansion protocols should be optimized for CD39/CD73 co-expression.

## Results

### Expanded human Tregs show enhanced suppressive function

**Fig. 1:**
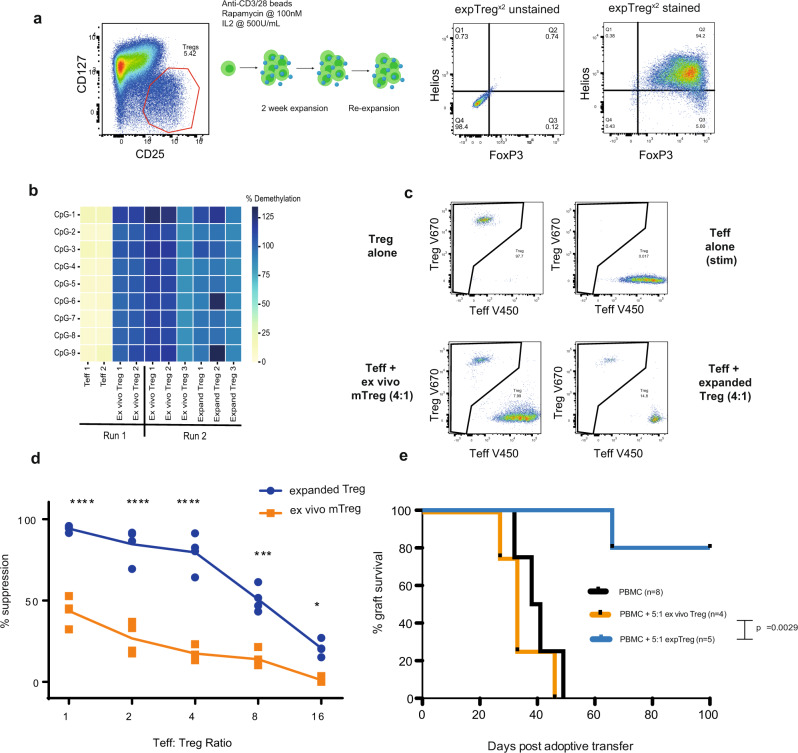
Therapeutically expanded human Tregs show enhanced suppressive function. **a** Flow-sorted human Tregs (CD3^+^CD4^+^CD25^hi^CD127^lo^) were expanded with anti-CD3/CD28 beads in presence of high-dose IL-2 (500 U/mL) and low-dose rapamycin (100 nM) in 2-week cycles. Representative plots showing Helios/FoxP3 expression on resting expTreg after two cycles of expansion (expTreg^x2^), gated on live CD4+ T cells. **b** *FOXP3* TSDR demethylation of flow-sorted human Tregs (CD3^+^CD4^+^CD25^hi^CD127^lo^) vs. T effectors (Teff), run 1 (*n* = 2) and ex vivo Treg vs. expanded Treg (expTregs), run 2 (*n* = 3, 2 female and 1 male donor). **c** Suppression assay labelling strategy: Flow-sorted ex vivo CD3^+^CD4^+^CD25^hi^CD127^lo^CD45RA^−^ memory Tregs (mTreg) or resting expTreg^x2^ were labelled with cell division dye V670 (top left plot), and sorted pan T (CD3^+^) effector cells with proliferation dye V450 (top right plot), then cultured separately or together (bottom left—ex vivo Tregs; bottom right, expTreg^×2^) in an in vitro Treg suppression assay ±anti-CD3/CD28 stimulation. **d** Summary showing the suppressive function of ex vivo mTregs (orange) vs. expTreg^×2^ (blue) from four donors at various Teff:Treg ratios. Line shows the mean percent suppression. *n* = cells from individual donors, analysed in independent experiments. Compared with ex vivo mTregs, expTreg^×2^ were more suppressive at all ratios tested (Two-way ANOVA with Bonferroni multiple testing correction *P < 0.05, **P < 0.01, ***P < 0.001, ****P < 0.0001). **e** In vivo skin graft model, in which mice received a skin graft plus 5 × 10^6^ allogeneic PBMCs together with 1 × 10^6^ ex vivo (orange) Tregs or 1 × 10^6^ resting expTregs^×2^ (blue). Data show a Kaplan–Meier survival curve of graft survival (Log-rank test, *p*-value as shown).

First we expanded flow-sorted CD4+ CD25^hi^CD127^lo^ human Tregs (Supplementary Fig. 1a) following a standard expansion protocol used widely for production of Treg cellular therapies (Fig. 1a), involving two (^×2^) 14-day cycles of stimulation with anti-CD3/CD28 beads, in the presence of high-dose IL-2 (500 U/mL) and low-dose rapamycin (100 nM). As demonstrated by full demethylation at the *FOXP3* TSDR (Fig. 1b) and lack of IL-2 production (Supplementary Fig. 1b) the sorted ex vivo CD4+ CD25^hi^CD127^lo^ Tregs were highly pure, and the resultant expTreg^x2^ maintained purity, as shown by HELIOS and FOXP3 co-expression (Fig. 1a), and retained *FOXP3* TSDR demethylation (Fig. 1b).

By day 14, expression of the proliferation marker Ki67 had returned to baseline (Supplementary Fig. 2a). Taken at this resting time point, expTreg^×2^ were found to be significantly more suppressive than their ex vivo counterparts in an in vitro suppression assay (Fig. 1c, d). CD4+ CD25^hi^CD127^lo^CD45RA^−^ ex vivo memory Tregs (mTregs) were selected as the comparator to better control for previous activation and memory status (Supplementary Fig. 2b) and stock pre-labelled allogeneic pan CD3^+^ Teffs were used as responders to allow for direct comparison.

In vivo, expTreg^×2^ were also significantly more suppressive than ex vivo CD4+ CD25^hi^CD127^lo^ Tregs when tested in a skin graft humanized mouse model (median survival time [MST] >100 days vs 33 days, *p* = 0.0029, Fig. 1e).

### ExpTregs acquire stable CD73 expression during expansion

**Fig. 2:**
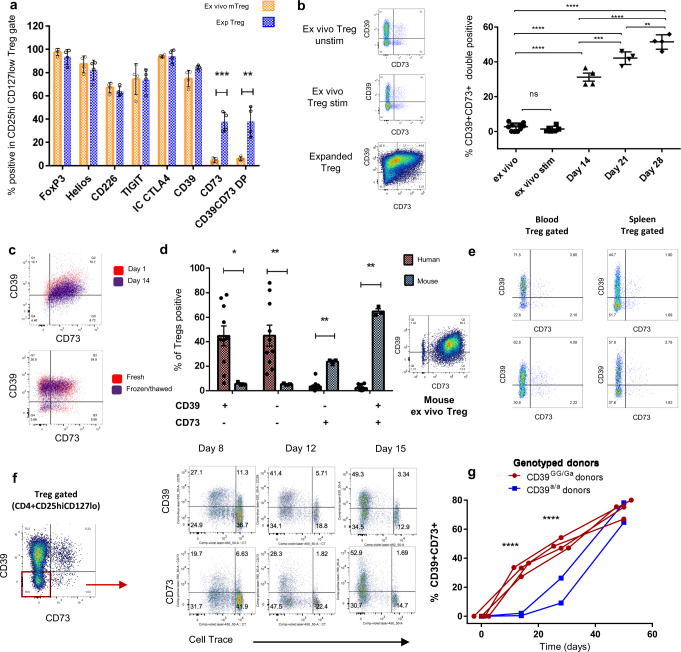
expTregs acquire stable CD73 expression and become CD39/CD73 co-expressing during their expansion. **a** Mean percent expression (±1SD) of Treg-associated markers expressed by resting expTreg^×2^ (blue bars) and ex vivo mTregs (orange bars) from four donors (multiple *t*-tests with Holm–Sidak correction for multiple comparisons). **b** Representative dotplots of CD39/CD73 co-expression in ex vivo Tregs, stimulated ex vivo Tregs and expTregs. Percentage expTreg CD39^+^CD73^+^ expression in ex vivo Tregs, stim ex vivo Tregs and expTregs from four donors over time (one-way ANOVA followed by Tukey’s multiple comparison testing). **c** Top plot: CD39/CD73 expression on flow-sorted expTregs rested for 1 day vs. expTregs rested for 2 weeks in low-dose IL-2 (50 U/mL). Bottom plot: CD39/CD73 expression on expTregs (red) vs. matched frozen/thawed expTregs (purple). **d** Percent of CD4^+^CD25^hi^FOXP3^+^ human (red, *n* = 10) vs. murine (blue, *n* = 3) Tregs that are CD39^+^CD73^−^, CD39^−^CD73^−^, CD39^−^CD73^+^ or CD39^+^CD73^+^. A representative dotplot of murine CD39/CD73 expression is shown (multiple unpaired *t*-tests with Holms Sidak correction for multiple comparisons). **e** CD39/CD73 expression on CD4^+^CD25^hi+^CD127^lo^ gated Tregs in matched blood and spleen from 2 deceased human donors. **f** Upregulation of CD39/CD73 on flow sorted, cell proliferation dye labelled CD39^−^CD73^−^ DN Tregs at day 8, 12 and 15 post-stimulation (representative plots from 3 donors). **g**: Percent CD39^+^CD73^+^ co-expression on expTregs from CD39^−^CD73^−^ DN Tregs from CD39 genotyped donors (CD39+^GG/Ga^ red, *n* = 4 and CD39lo^a/a^ blue, *n* = 2). (Two-way ANOVA with Bonferroni correction). *P < 0.05, **P < 0.01, ***P < 0.001, ****P < 0.0001. Summary data are presented as mean ± 1 SD. *n* represents cells from individual donors, analysed in independent experiments.

We next compared the expression of known Treg-associated molecules on resting expTreg^x2^ vs. ex vivo mTregs, including: FOXP3, HELIOS, TIGIT, CD226, CD39, CD73, CD25, CD127 and both intracellular and surface CTLA4. Since expTreg^×2^ were larger and more autofluorescent than ex vivo Tregs, the antibody panel and compensation matrix were optimized for each group separately (see Supplementary Fig. 3) and the percent positivity of each target antigen compared. Of these, only CD73 was differentially expressed (mean positivity 4.76% vs. 37.6%; *p* = 0.0003 Fig. 2a), and thus CD39 and CD73 co-expression was significantly higher in resting expTreg^×2^ compared to ex vivo mTregs (6.0% vs. 37.9%; *p* = 0.0027 Fig. 2a). Stimulation of total ex vivo Tregs (CD4 + CD25^hi^CD127^lo^) with anti-CD3/28 for 72 h did not increase CD73/CD39 co-expression; Fig. 2b shows the acquisition of CD39/CD73 double positivity by expTregs^×2^ over two cycles of expansion, compared to ex vivo Tregs and stimulated ex vivo Tregs. Acquisition of CD39/CD73 was similar for sorted naïve CD25^hi^CD127^lo^CD45RA^+^ (nTregs) and mTregs (Supplementary Fig. 4). CD73/CD39 co-expression was shown to be stable, surviving freeze/thaw and 2 weeks in culture with low-dose IL-2 and no additional stimulation (representative plots shown in Fig. 2c).

Although there is some conflict in the literature, in keeping with most reports we observed that ex vivo human Tregs expressed variable levels of CD39 and very little CD73, with only 2.4% (±2.3%) being double positive for CD73 and CD39. This is in sharp contrast to murine Tregs^1^, which expressed high levels of CD73 and CD39 constitutively (64.8 ± 3.5% CD39^+^/CD73^+^; Fig. 2d). As human studies typically involve blood, and murine studies spleen, we examined immune cells extracted from the spleen of two deceased human organ donors. This confirmed that both splenic and peripheral blood-derived human Tregs are largely CD73 negative, excluding a compartment effect (Fig. 2e).

Next, we flow-sorted highly pure CD39 and CD73 double negative Tregs from three donors, labelled them with cell division tracking dye and expanded them in culture as previously described. CD39 and CD73 expression increased over time with expansion, ruling out the possibility that expTreg CD39 and CD73 co-expression emerges due to the preferential expansion of small numbers of pre-existing CD39/CD73 double-positive cells. CD39 expression was gained first. By day 8 (equivalent to 2–3 cell divisions) approximately 40% of the starting CD39/CD73 double negative Tregs were positive for CD39, with 25% positive for CD73. By day 15 CD73 expression had increased in density and ~50% of the starting population had gained expression (Fig. 2f). By the end of the first cycle of expansion, ~30% of expTregs were CD39 and CD73 double positive, increasing to 50% by day 28 (Fig. 2g). Tregs expanded from donors with inherent low CD39 expression, correlating with a haplotype tagged by rs10748643^13,14^ (CD39lo^a/a^ donors, detailed in Supplementary Fig. 5), still acquired CD39 and CD73 co-expression, but this required additional rounds of expansion (Fig. 2g). This was due to delayed acquisition of CD39 rather than CD73 expression (Supplementary Fig. 5).

### ExpTregs gain the ability to convert extracellular ATP to immunosuppressive adenosine, explaining their increased suppressive function

**Fig. 3:**
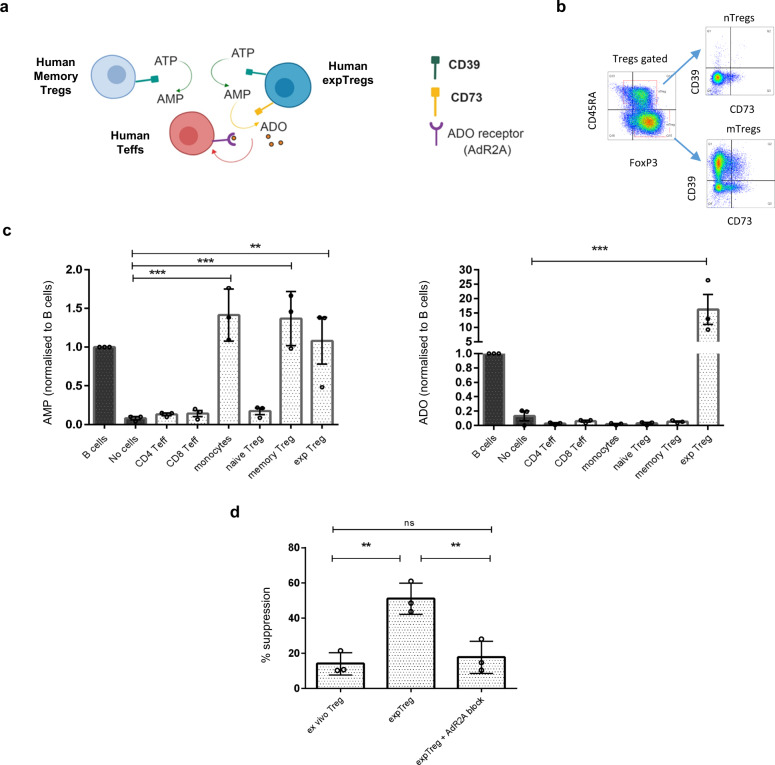
expTregs gain the ability to convert extracellular ATP to immunosuppressive adenosine, which explains their increased suppressive function. **a** Schematic showing extracellular ATP conversion to immunosuppressive adenosine (ADO): CD39 converts ATP to AMP then CD73 converts AMP to ADO, which acts via the adenosine receptor ADR2A on the surface of Teff cells. Created using Biorender.com. **b** Example dotplots showing differential expression of CD39 and CD73 in human ex vivo FOXP3^+^CD45RA^+^ (naive, nTregs) vs. FOXP3^+^CD45RA^−^ (memory, mTregs), **c** Mass spectrometric measurement of AMP and ADO in cell culture supernatants of various cell types from three donors following addition of 50 μM of ATP. Data shown are mean of area ratio normalized to matched B cells ±1 SD; one-way ANOVA with Dunnett’s multiple comparison against no cell control. In contrast to naive and memory ex vivo Tregs, expTreg^×2^ were able to convert ATP to ADO. **d** ExpTregs from three donors were cultured with Teffs at a ratio of 8:1 Teffs:Tregs in the absence or presence of ADR2A block ZM 241385 and the percentage suppression at day 5 compared with that of donor-matched ex vivo Tregs. Bar chart shows combined data for three donors ±1 SD (one-way ANOVA with Tukey’s multiple comparisons test) (*P < 0.05, **P < 0.01, ***P < 0.001, ****P < 0.0001). Summary data are presented as mean ± 1 SD. *n* represents cells from individual donors, analysed in independent experiments.

The coordinated action of the ectonucleotidases CD39 and CD73 converts ATP to immunosuppressive adenosine in a two-step process; firstly CD39 catalyses the hydrolysis of ATP to AMP, and secondly CD73 converts AMP to adenosine (ADO) (Fig. 3a). We confirmed the previously published observation that ex vivo nTregs are largely CD39/CD73 double negative^1,15,16^, in contrast, ex vivo mTregs are either CD39/CD73 double negative or CD39 single positive (shown by representative plots, Fig. 3b). To determine the functional consequences of CD39 and CD73 expression on the ability of Tregs to convert ATP to ADO, we exposed flow-sorted ex vivo nTregs, mTregs and expTreg^×2^ from three donors to 50 μM ATP, then measured AMP and ADO in the supernatant after 1 h by mass spectrometry. B cells (known to constitutively express CD39 and CD73^17^) and monocytes (known to be CD39 single positive^18^) as well as CD4 and CD8 Teffs were also assayed. In keeping with their expression patterns, B cells were the only ex vivo cell type tested able to produce both AMP and regulatory ADO (Fig. 3c). CD39 single-positive mTregs and monocytes were able to produce AMP from ATP, but were not able to convert AMP to ADO (Fig. 3c). In keeping with their increased co-expression of CD39/CD73, resting expTreg^×2^ were high ATP-to-adenosine converters (~16 fold higher than B cells; Fig. 3c).

To determine if increased production of ADO explains the enhanced suppressive action of expTregs, we co-cultured anti-CD3/CD28 activated Teffs, with resting expTreg^×2^ from three donors with and without adenosine receptor 2 A blockade (ADR2A being the adenosine receptor expressed by T cells^19,20^), and compared their suppressive function with ex vivo mTregs from the same donors. At a ratio of 8:1 Teffs:Tregs (chosen as a ratio where ex vivo mTregs are minimally suppressive, whereas expTreg^×2^ induce 50% suppression), ADR2A blockade significantly attenuated the suppressive effect of resting expTreg^×2^, returning their suppressive capacity to that obtained with donor-matched ex vivo mTregs (Fig. 3d and Supplementary Fig. 6). ADR2A blockade had no direct effect on Teff proliferation (Supplementary Fig. 6).

### ExpTregs undergo metabolic remodelling and utilize aerobic glycolysis during their expansion

**Fig. 4:**
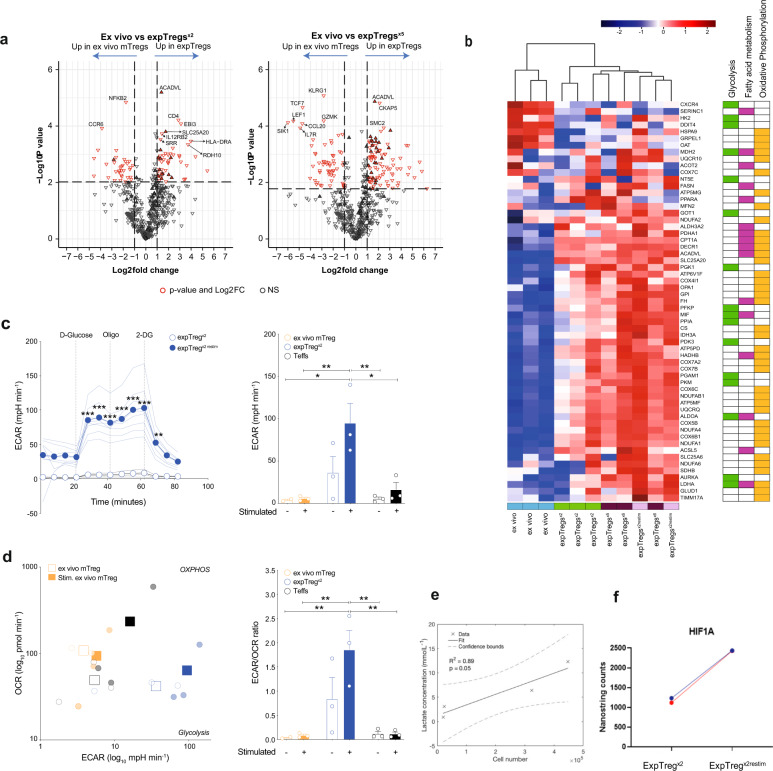
ExpTregs undergo metabolic remodelling and utilize aerobic glycolysis during their expansion. **a** Volcano plots of differential gene expression between ex vivo mTregs and resting expTregs (after 2 or 5/6 cycles) measured using Nanostring CAR-T panel. Gene Set Enrichment Analysis (GSEA) using the Hallmark pathways showed enrichment of metabolic genes (filled triangles). **b** Heatmap showing differential expression of genes involved in glycolysis, fatty acid metabolism and oxidative phosphorylation between ex vivo mTregs, resting expTregs (after 2 or 5/6 cycles) and re-stimulated expTregs (mid-cycle 3). **c** Representative glycolysis stress test of resting expTregs^×2^ and re-stimulated expTregs^×2restim^ from the same donor showing Extracellular Acidification Rate (ECAR) changes (left plot). Oligo oligomycin, 2DG 2-Deoxy-D-glucose. Single technical replicates (≥3) are shown as light lines, mean values are shown as dotted dark lines. Two-way ANOVA. Right plot shows histogram of ECAR values after D-Glucose in ex vivo mTregs, expTregs^×2^ and Teffs (unstimulated and stimulated). Single donors (≥2) are shown as circles, bar graphs show mean values ± SEM. One-way ANOVA. **d** Metabolic state of each group according to the Oxygen Consumption Rate (OCR) and ECAR after D-Glucose (left plot). Single donors (≥2) are shown as light circles, mean values are shown as dark squares. ECAR/OCR ratio as indicator of preferential use of glycolysis over OXPHOS (right plot). Single donors (≥2) are shown as circles, bar graphs show mean values ± SEM. One-way ANOVA. **e** Lactate in the cell supernatant of expTregs, measured spectrophotometrically, correlated positively with cell number (R^2^.0.89, *p* = 0.05). **f** HIF1A expression increased two-fold in expTreg^×2^ compared to matched ex vivo Tregs. (*P < 0.05, **P < 0.01, ***P < 0.001, ****P < 0.0001).

To further characterize how expTregs differ from ex vivo mTregs, transcriptomic analysis was performed using the Nanostring CAR-T Immune profiling panel, which quantifies the expression of 780 genes related to T-cell phenotype, activation and metabolism. RNA was extracted from ex vivo mTregs and from matched resting expTregs from three donors after either 2 (expTreg^×2^) or 5–6 cycles of expansion (expTreg^×5^). We detected 85 and 156 genes differentially expressed between ex vivo mTregs and matched resting expTreg^×2^ and expTreg^×5^, respectively (Fig. 4a and Supplementary data 1, greater than two-fold difference in expression, FDR *p* < 0.05). In expTregs, the majority of the differentially expressed genes were upregulated (51/85 and 97/156, ex vivo mTregs vs. expTreg^×2^ and expTreg^×5^, respectively). To assess if these genes clustered in a particular gene network, Gene Set Enrichment Analysis (GSEA) was performed using the Hallmark and Reactome gene sets and GO_Biological processes. These identified clear enrichment of metabolic processing in expTreg^×2^ and expTreg^×5^ that included oxidative phosphorylation, glycolysis and fatty acid metabolic pathways (Hallmark data shown in Fig. 4b). In addition, enrichment of genes relating to cell division and biosynthesis was evident (Supplementary data 2).

Next we took resting expTreg^×2^ and re-stimulated expTreg^×2restim^ (8 days post restimulation), and studied their metabolism using a glycolysis stress test with a XF Seahorse extracellular flux analyser. By assessing the extracellular acidification rates (ECAR) in response to D-Glucose, as an indicator of aerobic glycolysis, we found that expTreg^x2restim^ were significantly more glycolytic compared to unstimulated and stimulated ex vivo mTregs and Teffs (Fig. 4c). We then analysed the concomitant O_2_ consumption rate (OCR), as a marker of OXPHOS, to assess the metabolic state of each group (Fig. 4d). We found that the ECAR/OCR ratio was significantly higher in expTreg^x2restim^ compared to both unstimulated and stimulated ex vivo mTregs, as well as control Teffs (Fig. 4d). Overall, these data demonstrate that upon stimulation expTreg cells increase glycolysis, which they preferentially use over OXPHOS. In keeping, extracellular lactate (a by-product of glycolysis) was found to accumulate in the cell culture supernatant of expTregs, and this correlated with increasing expTreg numbers (Fig. 4e).

For two of the three donors studied, expTreg^×2restim^ were also available for Nanostring analysis. This demonstrated further increased expression of genes involved in oxidative metabolism and glycolysis (Fig. 4b), and the second most differentially expressed gene was the master transcriptional regulator of the adaptive response to hypoxia HIF1A, which doubled in expression upon restimulation (Fig. 4f; Supplementary data 1). No significant difference in HIF1A expression was seen between resting expTregs and ex vivo mTregs suggesting transient HIF1A upregulation during proliferation. Under hypoxic conditions—but also in the setting of aerobic glycolysis and mTORC1 signalling^21–23^ - HIF1A activates the transcription of over 100 genes including glucose transporters, glycolytic enzymes and also CD73 (*NT5E*)^24–27^ (reported HIF1A responsive genes captured by the CAR-T Nanostring panel are shown in Supplementary Fig. 7). This suggested a potential role for HIF1A signalling in CD73/*NT5E* upregulation in our metabolically active, proliferating expTregs.

### CD73 upregulation in human Tregs can be achieved by stabilization of HIF1A

**Fig. 5:**
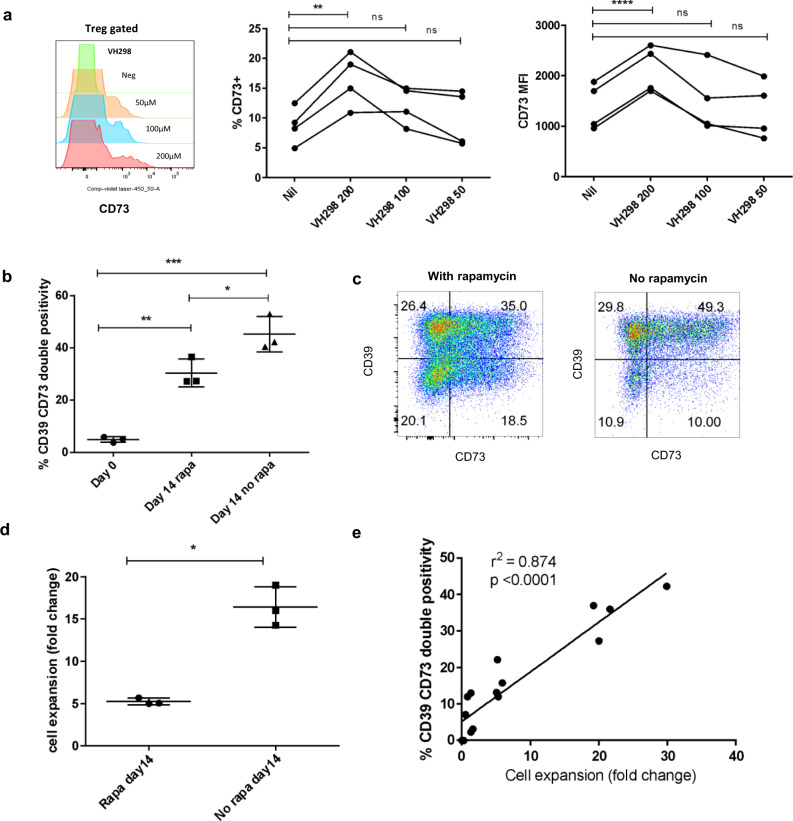
CD73 upregulation in human Tregs can be achieved by stabilization of HIF1A, and is gained more rapidly in the absence of rapamycin. **a** Percentage of CD73^+^ cells and MFI (geometric mean fluorescent intensity) of CD73 on ex vivo CD3^+^CD4^+^CD25^hi^CD127^lo^ Tregs from 4 donors as measured by flow cytometry following 30 h of culture with and without VH298 at 200, 100 and 50 μM. Increased CD73 was observed between the 200 μM concentration and untreated cells both for % CD73 and MFI. Example histogram from one donor shown on left. (One-way ANOVA with Dunnett’s correction). **b** Percentage CD39^+^CD73^+^ co-expression in expTregs cultured in the presence and absence of rapamycin after one round of expansion (three donors, one-way ANOVA followed by Tukey’s multiple comparison testing). **c** Representative example of CD39/CD73 co-expression in expTregs cultured with and without rapamycin. **d** Rate of expansion (fold change) in expTregs cultured with and without rapamycin (three donors, two-tailed paired *t*-test). **e** Rate of expansion vs gain of CD39/CD73 co-expression in expTregs (Pearson’s rank r = 0.875). *P < 0.05, **P < 0.01, ***P < 0.001, ****P < 0.0001.

Under resting/aerobic conditions HIF1A is constitutively degraded by the Von Hippel-Lindau (VHL) mediated ubiquitin protease pathway. Therefore, to determine if CD73 Treg expression could be induced by the post-translational stabilization of HIF1A, we cultured PBMCs for 30 h, in normoxic conditions (21% oxygen), with and without the addition of the VHL blocker VH298^28^. In the presence of VHL blockade, Treg CD73 expression increased significantly, with increases in both the percentage of CD73^+^ Tregs, and density of CD73 expression observed (Fig. 5a). Given the dependence of CD73 upregulation on HIF1A we hypothesized that Tregs expanded without rapamycin (an mTor and therefore HIF1A inhibitor) may upregulate CD73 more efficiently. In keeping with this, removing rapamycin increased the expansion rate and gain of CD39/CD73 co-expression in expTregs (Fig. 5b–e).

### CD39/CD73 co-expression is unrelated to loss of CD27 and gain of CD70

**Fig. 6:**
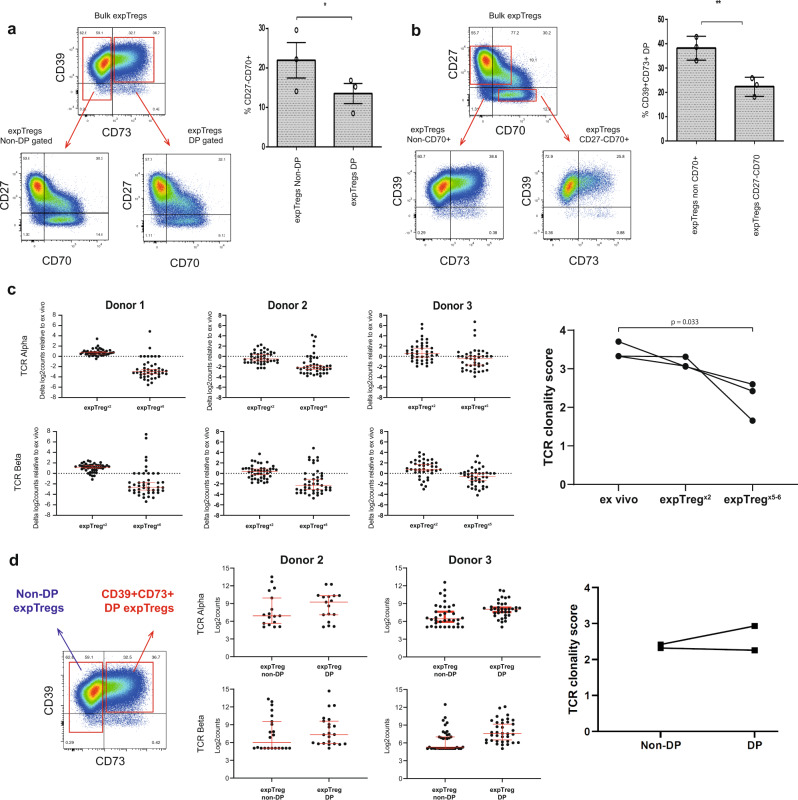
Multiple rounds of Treg expansion leads to reduced TCR diversity. **a** Analysis of CD27^−^CD70^+^ expTregs within gated CD39/CD73 double-positive expTregs and non-double-positive expTregs. Gating strategy is shown for one representative donor and summary data for three donors. **b** Analysis of CD39/CD73 co-expression within CD27^−^CD70^+^ expTregs and non-CD27^−^CD70^+^ expTregs. Gating strategy is shown for one representative donor and summary data for three donors. One-way ANOVA followed by Tukey’s multiple comparison testing. *P < 0.05, **P < 0.01, ***P < 0.001, ****P < 0.0001. **c** Expression of TCR alpha and TCR beta variable chains in expTreg^×2^ and expTreg^×5–6^ relative to their ex vivo mTreg counterpart (*n* = 3 donors, left plots. Red error bars show median with 95% CI, dotted green line denotes the mean of the ex *vivo* data set).TCR clonality is reduced in expTregs^×5–6^ as the mean expression of all alpha or beta chains is below zero, whereas only a few dominant TCR chains are expanded (values above zero). Log2 counts are shown in Supplementary Fig. 7. TCR clonality score of ex vivo mTregs vs. expTregs after 2 or 5–6 cycles of expansion (*n* = 3 donors, right plot) calculated using the expression of the variable TCR beta chain. **d** TCR clonality of sorted CD39^+^CD73^+^ double positive (DP Tregs) vs non-double-positive Tregs in two donors and TCR clonality score calculated using expression of the variable TCR beta chain.

We have previously reported that with prolonged Treg expansion a population of dysfunction Tregs emerge, characterized by loss of CD27 and gain of CD70 expression^29^. Therefore, we investigated the relationship between CD27, CD70, CD39 and CD73 expression on Tregs expanded for 5–6 cycles (expTreg^×5–6^) by flow cytometry. Although the CD27^−^CD70^+^ and CD39^+^CD73^+^ populations were not mutually exclusive, significantly fewer CD27^−^CD70^+^ cells were found within in the CD39/CD73 double-positive subpopulation compared to non-double-positive cells (Fig. 6a), and fewer CD39^+^CD73^+^ double-positive cells were found within the CD27^−^CD70^+^ gate compared to the remainder of the expTregs (Fig. 6b).

### Multiple rounds of Treg expansion leads to reduced TCR diversity

Given that individuals with inherent low CD39 expression require additional rounds of proliferation to gain CD39/CD73 co-expression, we determined how prolonged expansion might affect Treg TCR clonal diversity. TCR diversity was assessed by measuring the expression of the 45 TCR alpha and 46 TCR beta chain probes included in the Nanostring CAR-T panel on ex vivo mTregs and expTregs from the same donor. TCR diversity was similar between ex vivo mTregs and expTreg^×2^, but with additional expansion cycles (expTreg^×5–6^), TCR diversity decreased, suggesting the outgrowth of a few dominant clones (ex vivo vs. expTreg, *p* = 0.03, Fig. 6c). The specific alpha and beta chains that became dominant varied from donor to donor (Supplementary Fig. 7). To investigate the relationship between gain in CD39/CD73 co-expression and reduced clonal diversity in late expanded Tregs, we flow-sorted CD39/CD73 double-positive (DP) expTregs and non-double-positive (non-DP) expTregs—no difference in TCR clonality was seen between the two groups (Fig. 6d and Supplementary data 1).

## Discussion

In this study we have shown that human expTregs, expanded in vitro using a typical clinical expansion protocol (involving anti-CD3/CD28 stimulation in the presence of high-dose IL-2 and ±low-dose rapamycin), gain super-suppressive function in vitro and in vivo, at least in part due to the upregulation of CD73, leading to their enhanced ability to convert extracellular ATP to immunoregulatory adenosine. The result of our work has significant implications for therapeutic Treg expansion protocols and for the phenotypic testing of expTregs prior to adoptive transfer. As the number of trials testing Tregs increases, there is a desire amongst the community to develop a standardized framework for describing their isolation, generation, storage and phenotypic characterization prior to adoptive transfer^30^. We strongly suggest that CD39/CD73 co-expression is included in this “minimum information about Tregs” (“MITREG”) dataset.

Tregs are a subpopulation of CD4 T cells that are critical in maintaining peripheral self-tolerance. They are defined by surface expression of CD25 and low expression of CD127, neither of which are specific to Tregs, and by demethylation of the TSDR (which is considered to be the “gold-standard” for identifying a Treg) which leads to constitutive expression of their master transcription factor FOXP3^31^. FOXP3 controls many aspects of Treg biology, including their development, transcriptional programme and suppressive function^32^, and loss-of-function mutations in *FOXP3* cause lethal autoimmunity in both mice and humans^33,34^.

As we and others have previously reported^10–12^, expTregs were found to be super-suppressive when compared to matched ex vivo Tregs. This was demonstrated using a standard in vitro suppression assay and in a humanized mouse model of skin transplantation. Humanized mouse models are powerful pre-clinical tools for studying the in vivo effectiveness of human Treg populations^35,36^. In this study we co-transferred ex vivo and expTreg^×2^ at a ratio of 1:5 Tregs to PBMCs, a ratio selected based on our previous experience with this model, as a “sub-optimal” dose that would be most sensitive for demonstrating suppressive differences^10^. At this dose, ex vivo Tregs failed to enhance graft survival, whereas resting expTreg^x2^ led to long-term (>100 day) graft survival in 4 out of 5 animals tested.

Next, expression of Treg-associated markers was examined by flow cytometry. Using a predefined and therefore restricted panel, only CD73 was significantly upregulated on expTreg^×2^ vs. matched ex vivo mTregs (~8-fold change), leading to an increase in CD39/CD73 double-positive Tregs. Ex vivo mTregs were selected as the comparator to better control for previous activation and memory status. CD39, a member of the ectonucleoside triphosphate diphosphohydrolase (E-NTPDase) family, converts ATP to AMP, which is then hydrolyzed to immunoregulatory adenosine by CD73^37^. Accumulated extracellular adenosine then binds to its receptor (A2AR) on the surface of Teffs. Signals induced by A2AR increase intracellular cAMP, leading to inhibition of several cellular functions, such as T-cell proliferation and cytokine production^15,19^. Although there is some conflict in the literature^38^, it is generally accepted that human Tregs do not constitutively express CD73^1^, leading some to hypothesize that, in humans, adenosine-mediated suppression involving Tregs may depend upon paracrine mechanisms requiring the close proximity of CD39^+^Tregs to other cells expressing CD73 or other ectoenzymes^16,39^. Here we provide evidence for an additional/alternative explanation, i.e. that human Tregs do acquire the full enzymatic machinery to generate adenosine (as demonstrated here by mass spectrometry), but only after proliferation. In support of this, proliferating ICOS + Tregs in melanoma patients treated with high-dose IL-2 have been reported to co-express high levels of CD73 and CD39^40^. Failure to observe CD73 even on stimulated ex vivo Tregs may be an artefact of their anergy in vitro. Using an ADR2A blocker we confirmed that increased adenosine production was sufficient to explain the increased suppressive function of expTregs in vitro. Due to constraints imposed by low ex vivo Treg numbers, and to ensure sufficient technical repeats, this assay was only performed at one ratio; however, our data clearly demonstrate that production of immunosuppressive adenosine is an important regulatory mechanism gained by expanded Tregs. As this assay involved culturing Teffs with anti-CD3/CD28 beads, plus or minus the addition of Tregs, it confirms a direct effect on Teff cells, but may have missed additional enhanced suppressive mechanisms involving antigen presenting cells.

CD73 expression increased late, after several rounds of cell division, perhaps reflecting the importance of this pathway in switching off immune responses rather than preventing immune activation. However, once acquired, CD73 expression was found to be stable in vitro (at least up to 2 weeks) and to survive freeze/thawing. These are both important attributes when considering the practicalities of giving expTregs therapeutically. While performing these experiments, we observed high inter-individual variation in Treg CD39 expression, which has previously been reported^13,14^, and CD39 expression correlated with a haplotype tagged by rs10748643, with reduced CD39 expression from the A allele. Repeated stimulation and cell division could overcome this genotypic effect, but took up to 60 days in the CD39lo^a/a^ donors tested; this is in keeping with previous reports^41^. Given how common the rs10748643 A allele is globally (32.8% in Latino to 76.5% in East Asians^42^), CD39 genotype should be considered in therapeutic Treg expansion protocols, which may need to be extended in CD39lo^a/a^ individuals if full therapeutic gain is to be achieved. However, this will need to be balanced against the potential for exhaustion with repetitive rounds of stimulation^43^, including the emergence of a CD27^−^CD70^+^ dysfunctional population^29^ and against loss of clonal diversity. However, as shown, the CD73/CD39 double-positive population are neither enriched for CD27^−^CD70^+^ cells, nor less diverse than their non CD73/CD39 counterparts.

Transcriptome analysis of ex vivo mTregs and expTregs showed clear enrichment of genes involved in metabolic processing, including glycolysis, OXPHOS and fatty acid metabolism. OXPHOS involves intermediates from glucose, fatty acids, and amino acids, entering the oxygen-dependent tricarboxylic acid (TCA) cycle and electron transport chain (ETC) in mitochondria. In glycolysis, glucose is metabolized to pyruvate, which is converted into lactate rather than entering the TCA cycle. This is a rapid but less efficient way of producing ATP from glucose that is not dependent upon oxygen^44^; in addition it produces a pool of intermediate catabolites that can be utilized in other anabolic pathways, such as the pentose phosphate and serine pathways^45,46^. In keeping with this, GO term analysis demonstrated enrichment of biosynthetic pathways in expTregs. Although Nanostring is a highly sensitive and robust platform, as it is targeted we may have missed important changes in other pathways not captured by the panel.

Next, using a Seahorse XF extracellular flux analyser we assessed ECAR and OCR as surrogate measures of glycolysis and OXPHOS. In keeping with the literature, in response to activation Teffs upregulated both OXPHOS and glycolysis despite sufficient oxygen; aerobic glycolysis is well described in Teffs^47,48^. On the contrary, and in keeping with their anergic state in vitro, stimulated ex vivo mTregs did not significantly alter their metabolism. However, resting expTreg^×2^ demonstrated high levels of OXPHOS and aerobic glycolysis, and further shifted their metabolism to aerobic glycolysis on restimulation, resulting in the accumulation of lactate. Aerobic glycolysis is induced by the activation of the protein kinase B (Akt)/mammalian target of rapamycin complex 1 (mTORC1) signalling pathway, and by subsequent activation of the HIF1A which binds to response elements in target genes, including genes encoding glucose transporters and glycolytic enzymes^21–23^. In keeping with our data, HIF1A increases the abundance of lactate dehydrogenase (LDH), which catalyses the conversion of pyruvate to lactate, thereby limiting production of acetyl-CoA for the TCA cycle. It also increases pyruvate dehydrogenase kinase (PDK) leading to the inhibition of pyruvate dehydrogenase (PD) which converts pyruvate into acetyl-CoA.

While aerobic glycolysis is well described in activated Teffs, the role of glycolysis in Tregs is more contentious, with a number of recent papers challenging the dogma that Tregs depend largely on fatty acid oxidation^49,50^. For example, while Treg induction is enhanced by low mTOR signalling and inhibition of glycolysis^51–54^, mTORC1-deleted Tregs show impaired suppressive function in vitro and the mice developed a scurfy-like phenotype^55,56^ due to the loss of metabolic homeostasis in TCR-activated Tregs, reflected by reduced production of glucose-derived lipids and nucleotides and downregulated expression of mitochondrial genes^56–59^. Furthermore, glycolysis and HIF1A have been shown to be important in supporting the high metabolic demand of Treg migration, both in vitro and in vivo^60,61^. In addition to these predominantly murine studies, human Tregs have recently been shown to switch to glycolysis following TNF receptor 2 induced proliferation^62^, and recent proteomic analysis of human Tregs has provided further evidence of the importance of glycolysis in human Treg biology^63^. Taken together, our Nanostring and Seahorse data support the concept that, despite being cultured in low-dose rapamycin, rapidly expanding human Tregs undergo metabolic remodelling and a shift to aerobic glycolysis in response to TCR stimulation in the presence of IL-2, which sustains cell expansion, division and biosynthesis, as has been reported for Teffs.

By binding to a responsive element in the CD73 promotor, stabilized HIF1A has been reported to increase CD73 expression in human intestinal epithelial cells^24^ and breast cancer cell lines^25^, raising the possibility that increased expTreg CD73 expression may be intrinsically linked to their altered metabolic state, and potentially driven by HIF1A. To explore this we stabilized HIF1A using the VHL blocker, VH298. In normoxic/resting conditions HIF1A is constitutively degraded by the VHL mediated ubiquitin protease pathway. In contrast to several chemical agents known to stabilize HIF1A, such as Fe2^+^ substitutes and inhibitors of prolyl hydroxylase domain enzymes, VH298 is highly specific, capable of upregulating HIF1A-target genes, with no “off target” effects^28^. This confirmed that VHL blockade with VH298 was sufficient to increase expression of CD73 surface protein expression on human Tregs, with increases in both percentage positivity and density of expression. Interestingly, whilst glycolysis/HIF1A signalling is required to upregulate CD73, it appears expTregs do not need to be in a continued state of glycolysis to maintain CD73 at the cell surface.

Due to its relative specificity for suppressing the outgrowth of T effectors vs Tregs, the mTOR inhibitor rapamycin is commonly used to increase the purity of expTregs cultures. Our data demonstrate HIF1A drives acquisition of CD73 and therefore expTregs gain CD39/73 more rapidly in the absence of rapamycin, raising the question as to whether therapeutic Tregs should be expanded without rapamycin. However, as this will affect the purity of the final product, the decision to exclude rapamycin will depend on the purity of the starting population, for example it is likely to be required for magnetic bead isolated Tregs where the starting purity is relatively low.

With repeated rounds of expansion, required for gain of CD39/CD73 co-expression, TCR diversity reduced (although the CD39/CD73 double-positive subpopulation was no less diverse than the bulk population). Although Tregs can efficiently suppress polyclonal autoreactive responses through non-specific bystander suppression, and can recruit additional regulatory cell populations through “infectious tolerance”, it has been shown that TCR-restricted Tregs are less able to control murine GvHD, when compared to TCR-diverse Tregs^64^. In the same study, restricted Tregs were fully able to suppress anti-CD3 responses in vitro, suggesting that decreased in vivo suppression related to the probability of Treg activation, rather than a qualitative suppressive defect in the Tregs themselves. A limitation of our work is that Nanostring cannot assess true clonality, which can only be determined using the full sequence of the TCR, but rather estimates clonality as a function of TCR beta variable region expression.

In conclusion, using a standard therapeutic protocol, we have shown that human expTregs switch their metabolism from OXPHOS to aerobic glycolysis to meet the bioenergetic demands of expansion and proliferation. Associated HIF1A signalling promotes surface CD73 expression which, with CD39, provides expTregs with the full enzymatic machinery to convert ATP to immunoregulatory adenosine, leading to their enhanced suppressive function. We suggest that Treg expansion protocols should be optimized for CD39/CD73 co-expression and should take into account the additional time required for CD39lo^a/a^ individuals to gain expression if maximum therapeutic effect is to be achieved. This may need to be balanced against the need to retain clonal diversity.

## Methods

### Ethics statement

All work was completed under ethically approved studies. Human PBMCs were isolated from healthy volunteers after obtaining fully informed consent under CAMSAFE (REC-11/33/0007) or from buffy coats or leukocyte cones from healthy blood donors (NHSBT, UK). Human spleen was obtained from two deceased organ donors via the Cambridge Bioresource for Translational Medicine (CBTM) (REC 15/EE/0152 REC: East of England-Central Cambridge Research Committee). Healthy skin and blood was donated from patients undergoing plastic surgery procedures as previously described^36^ and with full informed consent under approval number 07/H0605/130 from the Oxfordshire Research Ethics Committee B. All mice were treated in strict accordance to the UK Animals (Scientific Procedures) Act of 1986 and under PPLs P8869535A or PPL80/8970.

### Cell isolation and magnetic separation

Human PBMC were isolated from heparinized blood, and splenic mononuclear cells (SMNCs) were isolated from mechanically dissociated and filtered human and murine spleens (from C57BL/6 mice) using Ficoll density gradient centrifugation (Ficoll PaquePlus; GE Healthcare, Amersham). CD19^+^ B cells, CD14^+^ monocytes and CD4^+^ T cells were isolated by positive magnetic selection (Miltenyi Biotec). Prior to flow sorting Tregs, untouched Pan T cells were enriched by negative selection using T-cell isolation kit II (Miltenyi biotec).

### Flow phenotyping and flow sorting

Cells were stained using a range of antibodies (BD, ebioscience, Biolegend) at 1:50 dilution unless otherwise stated, and blocked using 2% mouse serum (or 1% FCS for murine cells) in Facs buffer and/or Tru block monocyte blocker (Biolegend) as required. Intracellular staining was performed using the FOXP3 permeabilization staining kit (Invitrogen) for 45 min at room temperature followed by staining of intracellular markers in 1× perm wash buffer for 45 min at room temperature. Cell sorting was performed on either a FACS Aria (BD) or Influx (BD) cell sorter. Flow acquisition for phenotyping was performed on the Canto II or Fortessa LSR (BD Biosciences) and analysed using FlowJo v7.6.5-v10.0 (Tree Star Inc). Gating strategies and control staining are shown within each figure and additional control staining for CD73 and FOXP3 are shown in Supplementary Fig. 8.

### Ex vivo Treg isolation and expansion

Human ex vivo CD127^lo^CD25^+^CD4^+^ Tregs were flow sorted using a BD FACSAria cell sorter (BD Biosciences) after staining with: CD39-BB515 (clone Tu66, BD), CD25-PE (clone MA251, BD) or CD25-PE (BD), CD4 APCFire/750 (Clone A161A1, Biolegend) or CD4-ECD (Clone SFCI12T4D11, Beckman Coulter), CD73-BV421 (clone AD2, Biolegend), CD8 V500 (RPA-T8, BD), CD3-BV605 (SK7, Biolegend), CD127-AF647 (clone HIL-7R-M21, BD) or CD127-PEcy7 (eBioRDR5, ebioscience/Thermofisher) and CD45RA-BV786 (clone Hi100, BD), then expanded with 500–1000 U/mL human rIL2 (Miltenyi Biotec/Chiron) ±100 nM rapamycin (Miltenyi Biotec) and human Treg expansion kit anti-CD3/CD28 beads (Miltenyi Biotec/Invitrogen), using x-vivo 15 cell medium (Lonza) or RMPI-1640 medium supplemented with L-glutamine, penicillin-streptomycin (both PAA Laboratories, UK), sodium pyruvate (Gibco, UK) and 2–10% human AB pooled serum and media exchanged with fresh IL-2 every 3–5 days. For additional experiments, either memory Tregs (mTregs) were sorted as CD4^+^CD25^+^CD127^lo^CD45RA^−^, naïve Tregs were sorted as CD4^+^CD25^+^CD127^lo^CD45RA^+^ or CD39^−^CD73^−^ double negative Tregs were sorted as CD4^+^CD25^+^CD127^lo^CD39^−^CD73^−^. Beads were removed using MACSibead magnet (Milteny Biotec) prior to phenotyping or use in functional assays. Example sorts shown in Supplementary Figs. 1a and 2b).

### Measurement of *FOXP3* TSDR methylation

The methylation status of the *FOXP3* TSDR was measured using the protocol previously described^65^. In brief, expTregs were processed with the Qiagen Epitect Fast Bisulfite kit, which lyses the cells and performs the bisulfite conversion in a single step. First round PCR targeted 9 CpG sites within the *FOXP3* TSDR and a second round PCR added index sequences and illumina compatible ends allowing for sequencing on an Illumina MiSeq (2 × 300 bp reads). The percent FOXP3 demethylation was calculated as the proportion of sequencing reads containing 8 or 9 of the CpG sites within the TSDR being demethylated compared to the total number of sequencing reads. For female donors TSDR demethylation values were multiplied by two to account for the fact that one X chromosome is fully methylated due to X inactivation This required because FOXP3 is located on the X chromosome. Since there is a known bias in PCR efficiency of demethylated DNA^66,67^ this leads to values of over 100% in female donors.

### In vitro stimulation and suppression assays

For short term activation of sorted Tregs and Teffs, cells were cultured in RPMI complete media containing 10% FCS, Penicillin-streptomycin and Hepes ± anti-CD3/CD28 stimulation (Invitrogen dynabeads) at a cell:bead ratio of 4:1. In vitro suppression assays were performed as previously described^68^, briefly: Cell tracker V670 (invitrogen) labelled Tregs (ex vivo or expanded) and cell tracker V450 (Invitrogen) labelled MACS sorted Pan T cells (Miltenyi T-cell isolation kit II) were co-cultured in 96 V bottom plates (Greiner). Pan Teffs were seeded at 2 × 10^4^ cells per well in RPMI+ 3% human AB serum in the absence or presence of equal and titrated numbers of Tregs and stimulated using Miltenyi Tregs suppression inspector beads according to the manufacturer’s instructions. Stocks of allogeneic pan Teffs pre-labelled with V450 cell tracker dye were frozen in liquid nitrogen and used as standard Teffs for these assays. Assays were performed alone or in the presence of 2.5 μM ZM 241385 (Sigma)—a selective ADR2A antagonist. Proliferation was measured on day 4–5 using BD fortessa HTS plate reader. V670^+^ Tregs were gated out to enable analysis of the effector population (CD4^+^V450^+^V670^−^ cells) and suppression was calculated by taking the ratio between the proliferating and non-proliferating populations as previously described^68^.

### Skin allograft suppression assay model

Immunodeficient BALB/c Rag2^−/−^ IL2rg^−/−^ mice were purchased from Jackson Laboratories (Maine, USA) and housed under specific pathogen-free conditions in the Biomedical Services Unit at the John Radcliffe Hospital (Oxford, UK). Mice between ages of 6 and 12 weeks were used. Skin grafting was performed as previously described^36^. Briefly, 1 × 1 cm piece of human skin was fashioned and sutured to the mouse recipient skin on the left dorsal thorax over the costal margin. Grafts were left to heal for 35 days, before receiving an intraperitoneal injection of 5 × 10^6^ human peripheral blood mononuclear cells (PBMCs) allogeneic to the graft donor. Skin grafts were monitored regularly until loss. In experimental groups with Treg cells, 1 × 10^6^ Tregs from the same donor as PBMCs were co-injected with PBMCs. In all mice the degree of human leukocyte reconstitution was measured by flow cytometry at the time of harvest. Mice with human leukocyte chimerism levels of >0.1% in the blood or >1% in the spleen were defined as reconstituted and included in the study^36^. Skin allograft survival time was calculated from the point of PBMC injection to the point of complete graft loss/visible rejection.

### Functional metabolite assays (Mass Spectrometry)

The ability of different populations of cells to metabolize ATP to ADP, AMP and adenosine (and from AMP to adenosine) was measured using an ATP and AMP hydrolysis Mass Spectrometry protocol^69^. Briefly, cells of interest were isolated by either MACS or flow sorting and then separately cultured at 2 × 10^4^ cells/well in triplicate in 96-well plates at 37 °C in 5% CO_2_ for 1 h, in the presence or absence of 50 μM ATP or AMP. Cells were pelleted and supernatants flash frozen in liquid nitrogen and stored at −80 °C. Wells containing no cells and wells containing cells with no added ATP/AMP were used as controls. Supernatants were thawed and metabolites extracted using 2:1 methanol/chloroform followed by 1:1 water and chloroform. Nucleotides were measured by Liquid Chromatography tandem Mass Spectrometry (LC-MS/MS) using a Thermo Quantiva interfaced with a Ultra High performance Liquid Chromatography (UHPLC) Vanquish system (Thermo Scientific, Hemel Hempstead, UK). For chromatography on the UHPLC system, the strong mobile phase (A) was 100 mM ammonium acetate, the weak mobile phase was acetonitrile (B) and the LC column used was the ZIC-HILIC column from Sequant (100 × 2.1 mm, 5 µm). Relative abundance of each metabolite was measured as area under the curve at relevant masses and normalized to stable isotope labelled standards.

### Metabolism assays (Seahorse XF extracellular flux assay)

Oxygen consumption rates (OCR) and extracellular acidification rates (ECAR) were measured using a 96-well XF extracellular flux analyzer (EFA) (Seahorse Bioscience). Once isolated cells were seeded in assay medium (Agilent Seahorse XF RPMI Medium pH 7.4 cat no. 103576-100, containing 1 mM HEPES, and 2 mM Gibco Glutamax cat no. 35050-038) on a 96-well XF plate, pre-coated with Cell-Tak solution (Corning 354240, 3.5 ug/cm^2^ of surface area), at a concentration of 50,000/100,000 cells per well. After centrifugation at 200 × *g* for 1 min (zero braking), cells were left to rest in a 5% CO_2_ incubator at 37 °C for 30 min. Medium was exchanged and the plate was then incubated in a non-CO_2_ incubator at 37 °C for 1 h. A Glycolysis stress test was then performed by adding D-Glucose (final concentration 10 mM), oligomycin (final concentration 2.5 μM) and 2DG (final concentration 50 mM). After the run, cells were fixed with 4% PFA for 10 min and incubated with 1:10,000 DAPI. Data from were then normalized on the DAPI+ area averaged from 4 ROIs (×10 objective) for each well. Quality control for each well was performed on pH and oxygen flux, and wells that were not reliable were removed from further analysis, according to manufacturer’s instructions. The glycolytic values were calculated as the ECAR after addition of D-Glucose, while the OXPHOS values were calculated based on the OCR after addition of D-Glucose. The metabolic state of each group was calculated as the glycolytic value divided by the OXPHOS value.

### Lactate measurements

Extracellular lactate was measured in cell culture supernatants as a read out of glycolytic activity. Supernatants were collected at various timepoints during expTreg expansion and stored at −20 °C until required. Lactate was measured as previously described^68^, briefly: lactate was measured spectrophotometrically using a Dimension EXL autoanalyser (Siemens, Germany) with Flex© reagent cartridges (product code DF16). Lactate dehydrogenase (LDH, 40U), Nicotinamide adenine dinucleotide (NAD, 10 mmolL-1), dihydrazine sulphate (10 mmolL-1) and tris buffer (100 mmolL-1) were added to cell free expTreg expansion supernatants. The subsequent exchange of lactate to pyruvate, captured by the dihydrazine compound, is directly proportional to the change in NAD^+^ to NADH concentration measured at 340–383 nm, from which the initial lactate pool size was inferred.

### Transcriptomic profiling by Nanostring

100,000 freshly-sorted ex vivo mTregs and 100,000 resting, expanded Tregs from the same donors were harvested and washed twice in PBS, cells pelleted and lysed in RLT buffer (Qiagen). For the expanded Tregs, samples were taken after 2 rounds of expansion (expTreg^x2^) and after 5–6 rounds of expansion (expTreg^×5–6^). In addition, for 2 of the 3 donors, additional RNA was extracted mid-cycle 3, when >50% of the cells were in cycle (>50% Ki67^+^). RNA was extracted using Qiagen RNeasy micro kit and their transcriptome measured using the nanostring CAR-T characterization panel (Nanostring technologies) using an nCounter prep station and digital analyser; the Nanostring CAR-T panel measures the expression of 780 genes, that includes genes to characterize cell phenotype, activation state, metabolism, exhaustion and TCR diversity.

### Statistics and reproducibility

All flow cytometry data were analysed using FlowJo (version 10). Statistical tests were performed using Graphpad Prism 6.0 software (GraphPad Software Inc, California). Survival data were analyzed using log-rank tests. Comparisons between groups of three or more were compared using a one-way ANOVA with Tukey’s or Dunnet’s post-hoc multiple comparisons tests, as indicated. Comparisons of two groups were tested with either one-tailed or two-tailed Student’s *t*-tests, a two-way ANOVA with Bonferroni post-hoc multiple comparisons test, or multiple *t*-tests with Holm–Sidak post-hoc correction as appropriate. For Seahorse analysis comparisons between groups were calculated using one-way ANOVA followed by unpaired two-tailed Student’s *t*-tests. XF extracellular flux assay data were analysed by two-way ANOVA followed by Sidak’s multiple comparisons test, or one-way ANOVA followed by Tukey’s multiple comparison test, as appropriate. Comparisons over time were calculated using two-way ANOVA followed by Bonferroni’s multiple comparison tests. Lactate concentration was compared with expTreg number using a linear correlation (Data processed in Matlab 2017a, The Mathworks, MA). The number of biological replicates for each dataset are given in figure legend. All biological data points were derived from at least two technical replicates.

Nanostring data were normalized using nsolver 4.0 software (Nanostring), background thresholding was performed at the mean + 2SD of the negative probe values for each sample (range 15.2–36.4). Only probes with counts greater than the background threshold in at least two of the three samples were included in the analysis. Volcano plots were generated using the EnhancedVolcano R package^70^ and heatmaps were visualized with the Bioinfokit python package^71^. The Geneset enrichment analysis (GSEA) tool^72^ was used to determine if there was enrichment of any hallmark pathways^73^ between the ex vivo and expTregs. The Nanostring CAR-T panel contains probes to 45 TCR Alpha and 46 TCR beta variable chains, Briefly, the variable regions were assessed for overall expression and normalized to a panel standard, which allowed for more precise quantification. As an estimate of TCR diversity, a TCR clonality score was determined using the Shannon Diversity index calculation, a measure of species diversity in a community. This ecological measurement was used to account for the abundance and evenness of the T-cell beta variable regions present within a given sample versus the population of T-cell receptors within a given dataset. The score given is relative—a higher TCR score means a more diverse population of variable regions, and a less clonal population, whereas a lower score means less diversity, and a more clonal population.

### Genotyping

Donors were genotyped for SNP rs10748643, which is located within intron 1 of *ENTPD1*, the gene encoding CD39. Primers were designed that flank the variant (forward—GCACAGATGGTGTGCAGTCT, reverse—TCTTCCTGGCTCTCACACG), and PCR was performed to amplify this region using a 16 µl reaction containing 8 µl of Qiagen Multiplex PCR master mix, 0.5 µl of each forward and reverse primers (10 µM), 5 µl water and 2 µl of genomic DNA (5 ng/µl). The PCR cycling conditions consisted of 15 min hotstart at 95 °C, followed by 30 cycles of 95 °C for 30 seconds, 60 °C for 30 seconds and 72 °C for 30 seconds. The 97 bp PCR amplicon was digested with the restriction enzyme SCRF1 (New England Biolabs), and visualized on the Agilent Bioanalyser. The SCRF1 cuts the PCR amplicon to generate 90 and 7 bp products if homozygous for the A allele; 52, 38 and 7 bp products if homozygous for the G allele and 90, 52, 38 and 7 bp products if heterozygous (AG).

### Reporting summary

Further information on research design is available in the Nature Research Reporting Summary linked to this article.

## Supplementary information

Supplementary Information

Description of Additional Supplementary Files

Supplementary Data 1

Supplementary Data 2

Supplementary Data 3

Reporting Summary

## Data Availability

All raw nanostring data are available in supplementary 1 and 2. All source data used to generate figures are available in Supplementary Data 3. All other raw data are available from the corresponding author upon reasonable request.

## References

[1] Borsellino G (2007). Expression of ectonucleotidase CD39 by Foxp3+ Treg cells: hydrolysis of extracellular ATP and immune suppression. Blood.

[2] Wu DC (2013). Ex vivo expanded human regulatory T cells can prolong survival of a human islet allograft in a humanized mouse model. Transplantation.

[3] Nadig SN (2010). In vivo prevention of transplant arteriosclerosis by ex vivo-expanded human regulatory T cells. Nat. Med..

[4] Sagoo P (2011). Human regulatory T cells with alloantigen specificity are more potent inhibitors of alloimmune skin graft damage than polyclonal regulatory T cells. Sci. Transl. Med..

[5] Hippen KL (2011). Massive ex vivo expansion of human natural regulatory T cells (T(regs)) with minimal loss of in vivo functional activity. Sci. Transl. Med..

[6] Sawitzki B (2020). Regulatory cell therapy in kidney transplantation (The ONE Study): a harmonised design and analysis of seven non-randomised, single-arm, phase 1/2A trials. Lancet.

[7] Romano M, Fanelli G, Albany CJ, Giganti G, Lombardi G (2019). Past, present, and future of regulatory T cell therapy in transplantation and autoimmunity. Front. Immunol.

[8] Fraser H (2018). A rapamycin-based GMP-compatible process for the isolation and expansion of regulatory T cells for clinical trials. Mol. Ther. Methods Clin. Dev..

[9] Battaglia M, Stabilini A, Roncarolo M-G (2005). Rapamycin selectively expands CD4 + CD25 + FoxP3+ regulatory T cells. Blood.

[10] Issa F (2019). Transiently activated human regulatory T cells upregulate BCL-XL expression and acquire a functional advantage in vivo. Front. Immunol..

[11] Canavan JB (2016). Developing in vitro expanded CD45RA^+^ regulatory T cells as an adoptive cell therapy for Crohn’s disease. Gut.

[12] Cao T, Wenzel SE, Faubion WA, Harriman G, Li L (2010). Enhanced suppressive function of regulatory T cells from patients with immune-mediated diseases following successful ex vivo expansion. Clin. Immunol..

[13] Orrù V (2013). Genetic variants regulating immune cell levels in health and disease. Cell.

[14] Friedman DJ (2009). From the Cover: CD39 deletion exacerbates experimental murine colitis and human polymorphisms increase susceptibility to inflammatory bowel disease. Proc. Natl Acad. Sci. USA.

[15] Deaglio S (2007). Adenosine generation catalyzed by CD39 and CD73 expressed on regulatory T cells mediates immune suppression. J. Exp. Med.

[16] Dwyer KM (2010). Expression of CD39 by human peripheral blood CD4+ CD25+ T cells denotes a regulatory memory phenotype. Am. J. Transplant..

[17] Saze Z (2013). Adenosine production by human B cells and B cell–mediated suppression of activated T cells. Blood.

[18] Pulte ED (2007). CD39/NTPDase-1 activity and expression in normal leukocytes. Thromb. Res..

[19] Koshiba M (1999). S. Patterns of A2A extracellular adenosine receptor expression in different functional subsets of human peripheral T cells. Flow cytometry studies with anti-A2A receptor monoclonal antibodies. Mol. Pharmacol..

[20] Lukashev DE (2003). Analysis of A2a receptor-deficient mice reveals no significant compensatory increases in the expression of A2b, A1, and A3 adenosine receptors in lymphoid organs. Biochem. Pharmacol..

[21] Cheng S-C (2014). mTOR- and HIF-1α-mediated aerobic glycolysis as metabolic basis for trained immunity. Science.

[22] Semenza GL, Roth PH, Fang HM, Wang GL (1994). Transcriptional regulation of genes encoding glycolytic enzymes by hypoxia-inducible factor 1. J. Biol. Chem..

[23] Del Rey MJ (2017). Hif-1α knockdown reduces glycolytic metabolism and induces cell death of human synovial fibroblasts under normoxic conditions. Sci. Rep..

[24] Synnestvedt K (2002). Ecto-5’-nucleotidase (CD73) regulation by hypoxia-inducible factor-1 mediates permeability changes in intestinal epithelia. J. Clin. Invest..

[25] Samanta D (2018). Chemotherapy induces enrichment of CD47+/CD73+/PDL1+ immune evasive triple-negative breast cancer cells. Proc. Natl Acad. Sci. USA.

[26] Oki S (2018). ChIP-Atlas: a data-mining suite powered by full integration of public ChIP-seq data. EMBO Rep..

[27] Slemc L, Kunej T (2016). Transcription factor HIF1A: downstream targets, associated pathways, polymorphic hypoxia response element (HRE) sites, and initiative for standardization of reporting in scientific literature. Tumour Biol..

[28] Frost J (2016). Potent and selective chemical probe of hypoxic signalling downstream of HIF-α hydroxylation via VHL inhibition. Nat. Commun.

[29] Arroyo Hornero R (2020). CD70 expression determines the therapeutic efficacy of expanded human regulatory T cells. Commun. Biol.

[30] Fuchs A (2018). Minimum information about T regulatory cells: a step toward reproducibility and standardization. Front. Immunol.

[31] Polansky JK (2008). DNA methylation controls Foxp3 gene expression. Eur. J. Immunol..

[32] Fontenot JD, Gavin MA, Rudensky AY (2003). Foxp3 programs the development and function of CD4+CD25+ regulatory T cells. Nat. Immunol..

[33] Bacchetta R (2006). Defective regulatory and effector T cell functions in patients with *FOXP3* mutations. J. Clin. Invest..

[34] Brunkow ME (2001). Disruption of a new forkhead/winged-helix protein, scurfin, results in the fatal lymphoproliferative disorder of the scurfy mouse. Nat. Genet..

[35] Adigbli, G. et al. Humanization of immunodeficient animals for the modeling of transplantation, graft versus host disease and regenerative medicine. *Transplantation* 1583–1599 (2020). 10.1097/TP.0000000000003177 PMC7590965 32068660

[36] Issa F (2010). Ex vivo–expanded human regulatory T cells prevent the rejection of skin allografts in a humanized mouse model. Transplantation.

[37] Faas MM, Sáez T, de Vos P (2017). Extracellular ATP and adenosine: the Yin and Yang in immune responses?. Mol. Asp. Med..

[38] Alam MS (2009). CD73 is expressed by human regulatory T helper cells and suppresses proinflammatory cytokine production and Helicobacter felis-induced gastritis in mice. J. Infect. Dis..

[39] Mandapathil M (2010). Generation and accumulation of immunosuppressive adenosine by human CD4^+^ CD25^high^ FOXP3^+^ regulatory T cells. J. Biol. Chem..

[40] Sim GC (2014). IL-2 therapy promotes suppressive ICOS^+^ Treg expansion in melanoma patients. J. Clin. Invest..

[41] Rissiek A (2015). The expression of CD39 on regulatory T cells is genetically driven and further upregulated at sites of inflammation. J. Autoimmun..

[42] Karczewski KJ (2020). The mutational constraint spectrum quantified from variation in 141,456 humans. Nature.

[43] Hoffmann P (2009). Loss of FOXP3 expression in natural human CD4+CD25+ regulatory T cells upon repetitive in vitro stimulation. Eur. J. Immunol..

[44] Pfeiffer T, Schuster S, Bonhoeffer S (2001). Cooperation and competition in the evolution of ATP-producing pathways. Science.

[45] Wang R (2011). The transcription factor Myc controls metabolic reprogramming upon T lymphocyte activation. Immunity.

[46] Ma EH (2017). Serine is an essential metabolite for effector T cell expansion. Cell Metab..

[47] Frauwirth KA (2002). The CD28 signaling pathway regulates glucose metabolism. Immunity.

[48] Newsholme EA, Crabtree B, Ardawi MS (1985). The role of high rates of glycolysis and glutamine utilization in rapidly dividing cells. Biosci. Rep..

[49] Angelin A (2017). Foxp3 reprograms T cell metabolism to function in low-glucose, high-lactate environments. Cell Metab..

[50] Hashimoto H, McCallion O, Kempkes RWM, Hester J, Issa F (2020). Distinct metabolic pathways mediate regulatory T cell differentiation and function. Immunol. Lett..

[51] Shi LZ (2011). HIF1alpha-dependent glycolytic pathway orchestrates a metabolic checkpoint for the differentiation of TH17 and Treg cells. J. Exp. Med.

[52] Li W (2019). Targeting T cell activation and lupus autoimmune phenotypes by inhibiting glucose transporters. Front. Immunol.

[53] Delgoffe GM (2009). The mTOR kinase differentially regulates effector and regulatory T cell lineage commitment. Immunity.

[54] Delgoffe GM (2011). The kinase mTOR regulates the differentiation of helper T cells through the selective activation of signaling by mTORC1 and mTORC2. Nat. Immunol..

[55] Sun I-H (2018). mTOR complex 1 signaling regulates the generation and function of central and effector Foxp3+ regulatory T cells. J. Immunol..

[56] Zeng H (2013). mTORC1 couples immune signals and metabolic programming to establish T reg -cell function. Nature.

[57] Chapman NM (2018). mTOR coordinates transcriptional programs and mitochondrial metabolism of activated T reg subsets to protect tissue homeostasis. Nat. Commun..

[58] Chapman NM, Chi H (2015). mTOR links environmental signals to T cell fate decisions. Front. Immunol.

[59] Field CS (2020). Mitochondrial integrity regulated by lipid metabolism is a cell-intrinsic checkpoint for Treg suppressive function. Cell Metab..

[60] Kishore M (2017). Regulatory T cell migration is dependent on glucokinase-mediated glycolysis. Immunity.

[61] Miska J (2019). HIF-1α is a metabolic switch between glycolytic-driven migration and oxidative phosphorylation-driven immunosuppression of Tregs in glioblastoma. Cell Rep..

[62] de Kivit, S. et al. Stable human regulatory T cells switch to glycolysis following TNF receptor 2 costimulation. *Nat. Metab*. 10.1038/s42255-020-00271-w (2020). 10.1038/s42255-020-00271-w 32958937

[63] Procaccini C (2016). The proteomic landscape of human ex vivo regulatory and conventional T cells reveals specific metabolic requirements. Immunity.

[64] Föhse L (2011). High TCR diversity ensures optimal function and homeostasis of Foxp3+ regulatory T cells. Eur. J. Immunol..

[65] Rainbow DB (2015). Epigenetic analysis of regulatory T cells using multiplex bisulfite sequencing. Eur. J. Immunol..

[66] Moskalev EA (2011). Correction of PCR-bias in quantitative DNA methylation studies by means of cubic polynomial regression. Nucleic Acids Res..

[67] Warnecke PM (1997). Detection and measurement of PCR bias in quantitative methylation analysis of bisulphite-treated DNA. Nucleic Acids Res..

[68] Grist, J. T. et al. Extracellular lactate: a novel measure of T cell proliferation. *J. Immunol*. 10.4049/jimmunol.1700886 (2017). 10.4049/jimmunol.1700886 PMC5776880 29288205

[69] West JA (2016). A targeted metabolomics assay for cardiac metabolism and demonstration using a mouse model of dilated cardiomyopathy. Metabolomics.

[70] Blighe, K. *kevinblighe/EnhancedVolcano*. https://github.com/kevinblighe/EnhancedVolcano (2020).

[71] Bedre, R. reneshbedre/bioinfokit: Bioinformatics data analysis and visualization toolkit. *Zenodo*10.5281/zenodo.3841708 (2020).

[72] Subramanian A (2005). Gene set enrichment analysis: a knowledge-based approach for interpreting genome-wide expression profiles. Proc. Natl Acad. Sci. USA.

[73] Liberzon A (2015). The molecular signatures database hallmark gene set collection. Cell Syst..

