# Supplementary material for: Therapeutically expanded human regulatory T-cells are super-suppressive due to HIF1A induced expression of CD73
Source: Commun Biol. 2021 Oct 14;4:1186. doi: 10.1038/s42003-021-02721-x (PMC8516976; doi:10.1038/s42003-021-02721-x)
Supplement: Supplementary file 1 — Supplementary Information [file 42003_2021_2721_MOESM1_ESM.pdf]

Supplementary Figures

a: Flow sort for ex vivo CD25hiCD127low Tregs

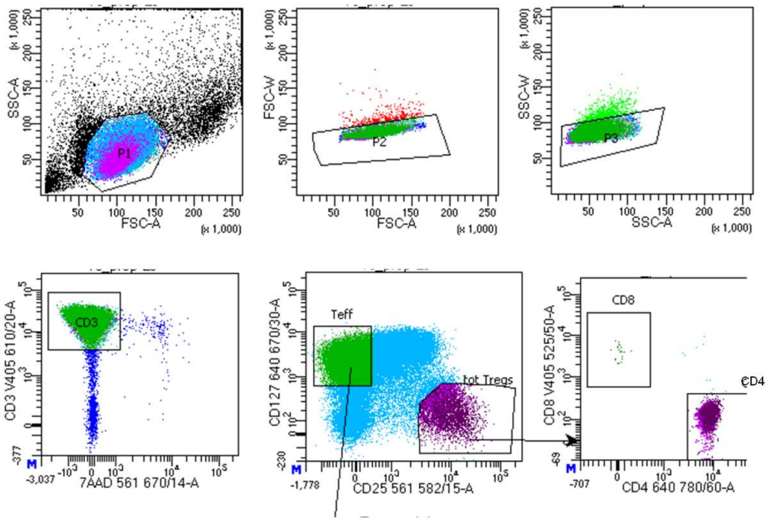

b: IL2 production by ex vivo Teffectors and Tregs – intracellular cytokine staining

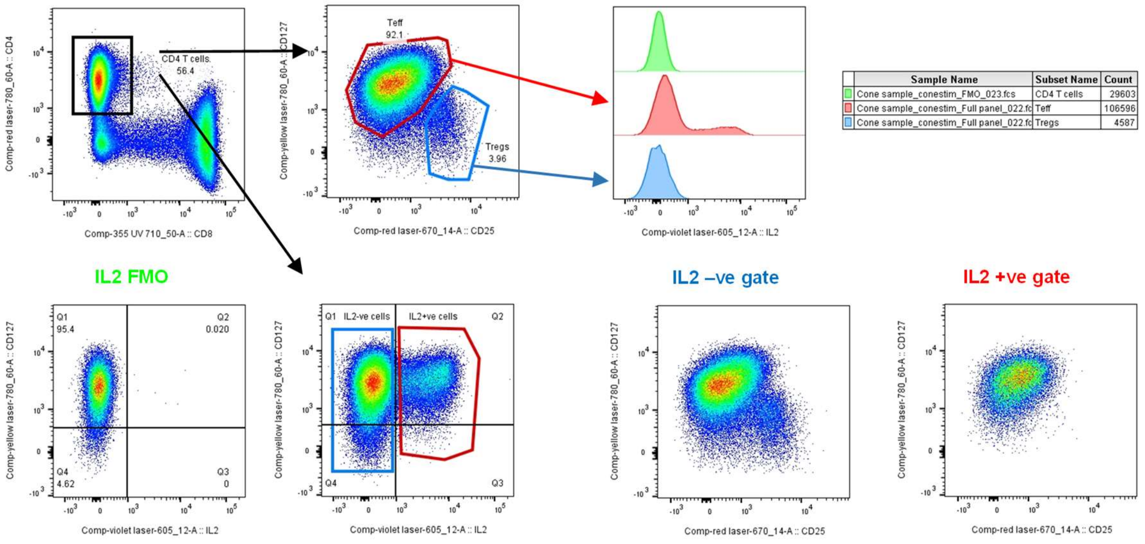

Supplementary Figure 1:

a: Sort of ex vivo CD25^{hi}CD127^{lo} Tregs

Representative example showing flow sort of Teff and Tregs based on CD127 and CD25.

b: IL2 Production by Teffs and Tregs

Representative example showing intracellular IL2 staining of stimulated PBMC (PMA/ionomycin, 6 hours). IL2 production is confined to the CD127+CD25- “Teffector” gate whereas CD25hi CD127lo Tregs do not make IL2.

a: Ki67 expression mid- and end- of expansion

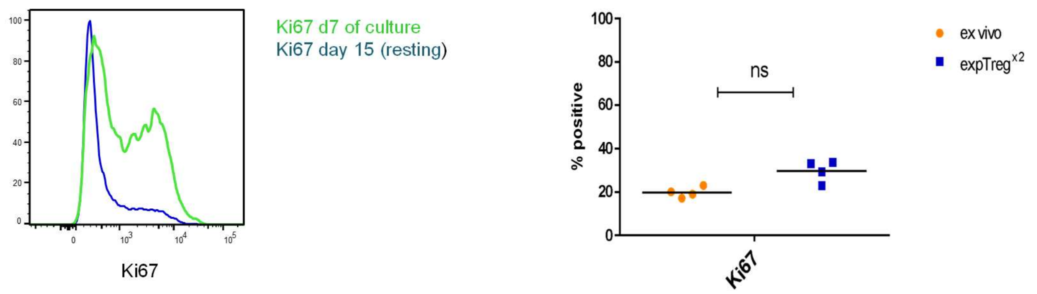

b: Representative flow sort for naïve (nTreg) and memory (mTreg) Tregs

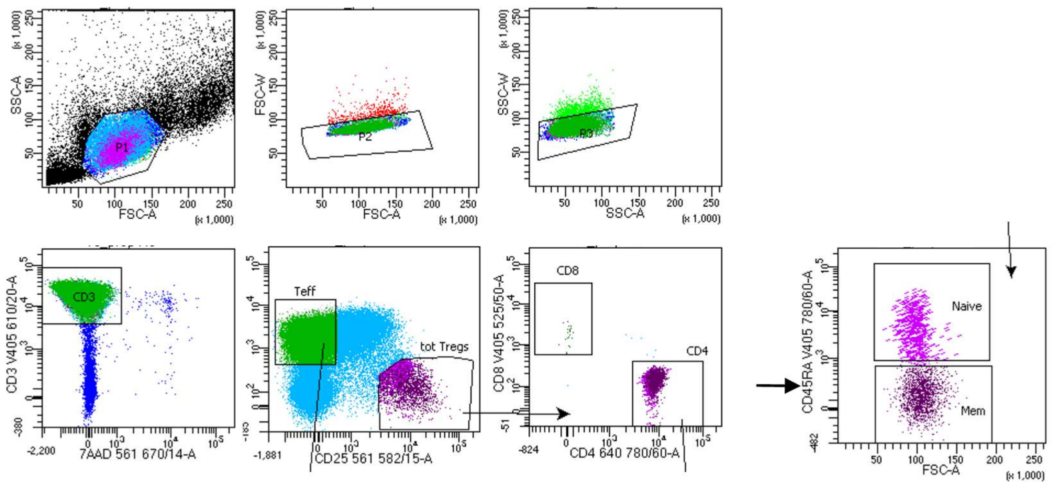

Supplementary Figure 2:

a: ExpTregs return to resting state at end of expansion cycle

Representative example comparing Ki67⁺ proliferating expTregs mid-cycle at day 7 (green) with Tregs at the end of the expansion cycle at day 15 (blue), left hand plot. Ki67 expression was not significantly different between *ex vivo* mTreg and expTreg^{x2}, right plot (n = 4 separate donors) in a two-tailed paired Student's t-test.

b: Representative example showing sort of naïve (n-) and memory (m-) Tregs based on CD127low/CD25 high and CD45RA expression.

a Gating strategy – Ex vivo Treg

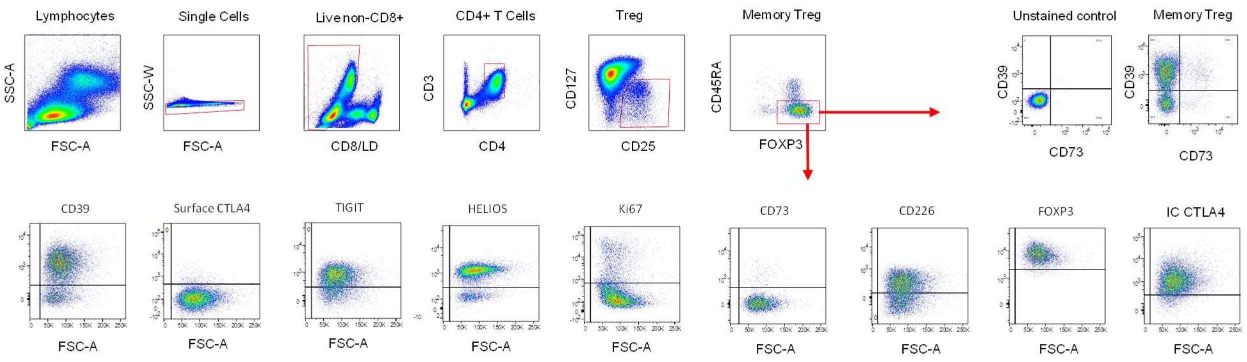

b Gating strategy – Expanded Treg

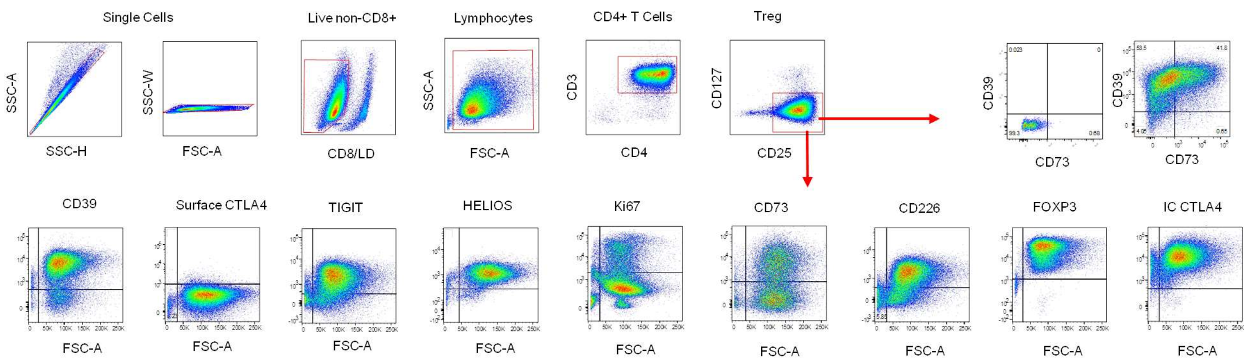

c

Panel for Treg Phenotyping:

SURFACE		Cat no	Clone	INTRACELLULAR		Cat no	Clone
CTLA-4	BV421	BL 369606	BNI3	HELIOS	FITC	BL 137214	22F6
CD3	BV570	BL 300436	UCHT1	CTLA-4	PE-Dazzle	BL 369616	BNI3
CD8	v500	BD 560774	RPA-T8	FOXP3	PE	eBio 12-4776-41	PCH101
CD45RA	BV605	BL 304134	HI100	FOXP3	PE	BL 320207	259D
CD39	BV650	BD 563681	Tu66	Ki67	BUV395	BD 564071	B56
CD73	BV786	BL 344028	AD2				
TIGIT	PerCP-efl710	eBio 46-9500-42	MBSA43				
CD127	PE-Cy7	eBio 25-1278-42	eBioRDR5				
CD25	APC		BD 2A3				
CD25	APC		MA251				
CD4	APC-R700	BD 564976	RPA-T4				
CD226	APC-Fire750	BL 338320	11A8				

Supplementary Figure 3:

Gating strategy and antibody panel used for *ex vivo* and expTreg phenotyping

a: Gating strategy and representative example of flow cytometric analysis of Treg markers on *ex vivo* mTregs. PBMC were stained by flow cytometry and live $CD3^{+}CD4^{+}CD25^{hi}CD127^{lo}CD45RA^{-}$ Tregs were gated. Values in the top right quadrants were used as % positivity.
b: Gating strategy and representative example of flow cytometric analysis of Treg markers on expTregs. Values in the top right quadrants were used as % positivity.
c: The Treg phenotyping flow panel showing all antibodies and clones.
PBMC and expTregs from the same donors were frozen, thawed and stained on the same day using the same antibody panel.

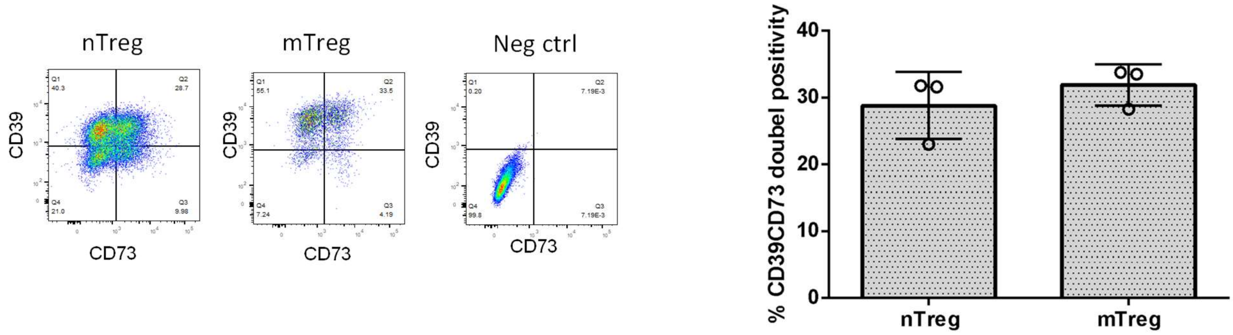

Supplementary Figure 4:

Acquisition of CD39 and CD73 by Tregs expanded from naïve and memory ex vivo Tregs

Representative staining at day 21 post-expansion of nTreg vs mTreg from 1 donor (left plots) and summary data comparing % CD39/CD73 co-expression in Tregs expanded from nTreg vs mTreg in 3 donors (barchart, right shows mean % double positivity +/- 1 SD).

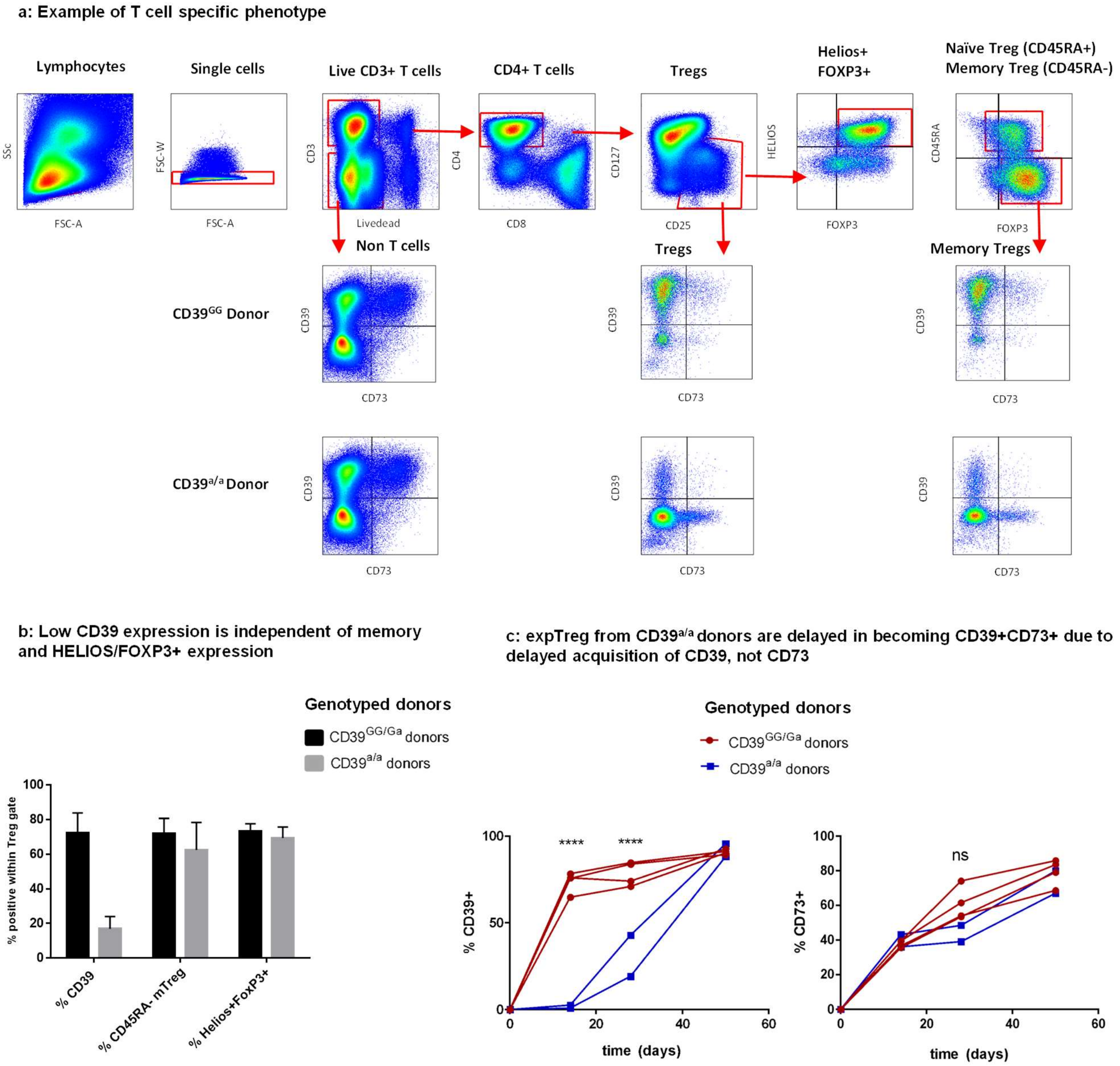

Supplementary Figure 5:

CD39lo$^{a/a}$ Individuals have inherently low T cell expression of CD39

a: Representative gating strategy used for analysing CD39^{+} (CD39+$^{GG/Ga}$) and CD39low (CD39lo$^{a/a}$) donors, demonstrating T cell specific loss of CD39 expression. CD39+$^{GG/Ga}$ and CD39lo$^{a/a}$ donors express normal levels of CD39 on non-T cells (left lower dot plots) but CD39lo$^{a/a}$ donors have reduced levels on Tregs (middle dot plot). Low CD39 is not due to differences in proportion of memory Tregs, since memory (CD45RA^{-}FOXP3^{+}) Tregs also lack CD39 in CD39lo$^{a/a}$ donors (right lower dot plots).

b: CD39 expression in CD39lo$^{a/a}$ donors is independent of memory and CD39lo$^{a/a}$ donors have similar proportion of HELIOS^{+}FOXP3^{+} double positive Tregs as shown by summary data of 3 CD39+$^{GG/Ga}$ and 3 CD39lo$^{a/a}$ donors, analysed by flow cytometry (bar chart shows mean % positivity within CD3^{+}CD4^{+}CD25^{hi}CD127^{lo} Treg gate +/- 1 SD).

c: Comparison of CD39 expression (left) and CD73 expression (right) over time on expanded CD39^{-}CD73^{-} DN Tregs from CD39+$^{GG/Ga}$ and CD39lo$^{a/a}$ donors, demonstrating delayed acquisition of CD39 but not CD73. (Two way ANOVA with Bonferroni multiple testing correction).

*P <0.05, **P <0.01, ***P <0.001, ****P <0.0001.

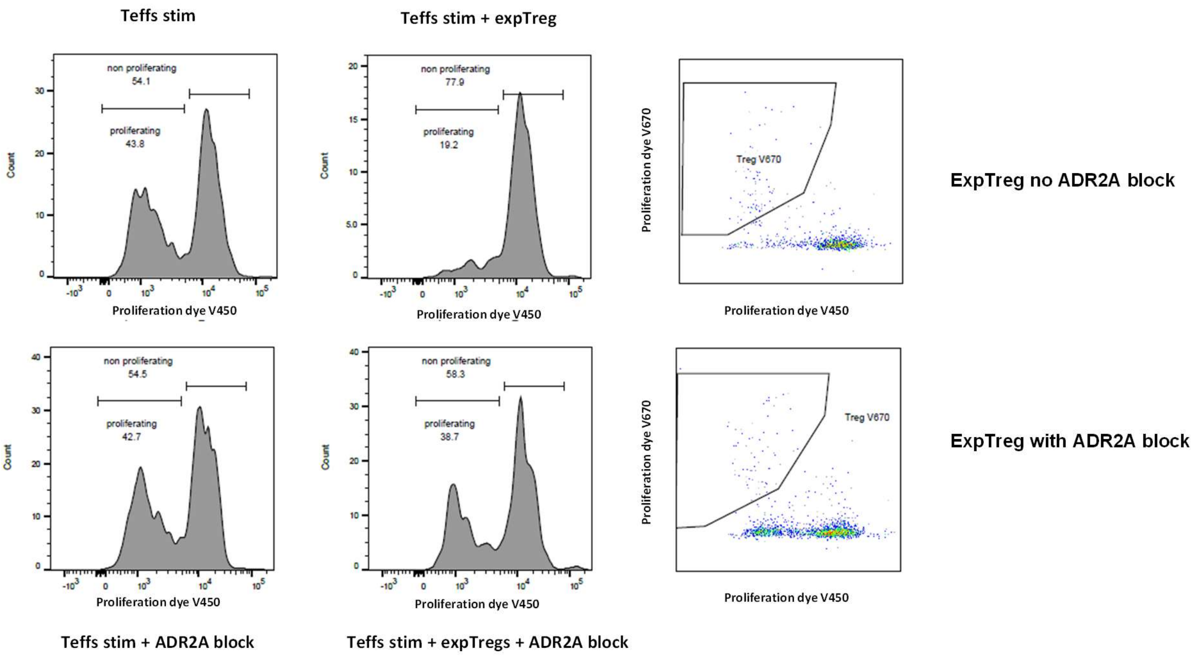

Supplementary Figure 6:
Example ADR2A blocking assay
Representative flow plot of AD2RA blocking suppression assay. V450 proliferation dye labelled Teffector cells (Teffs) were stimulated for 5 days using anti-CD3/CD28 Treg suppression assay beads with (middle histograms) and without (left histograms) expTregs at a ratio of 8:1 Teffs:Tregs and with (bottom plots) and without (top plots) ADR2A block. Histograms show V450 dye dilution of the Teffs (V670- non Treg) cells as demonstrated by the gating in right hand dot plots.

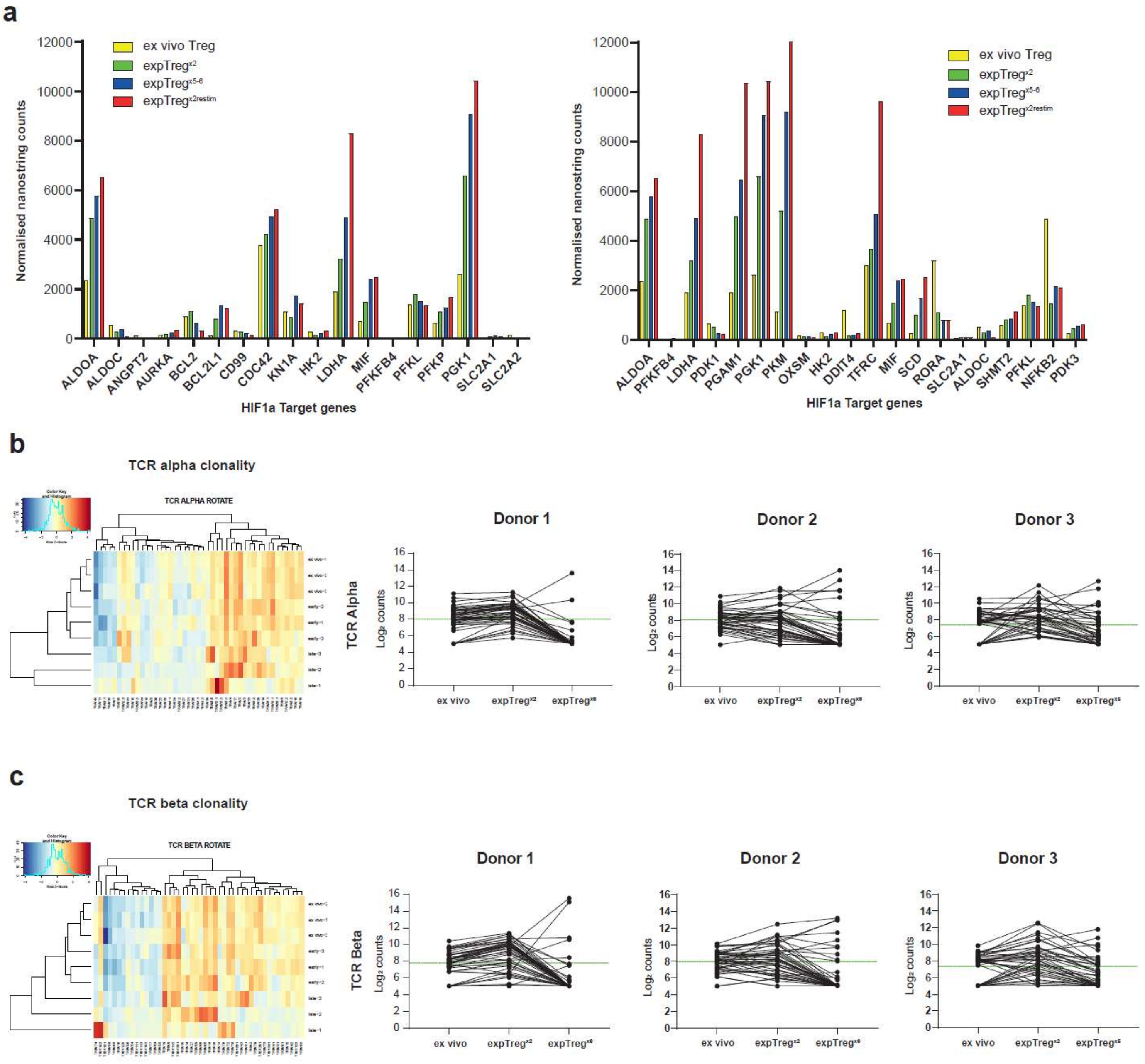

Supplementary Figure 7:
Additional Nanostring data

a: Expression levels of HIF1A responsive genes captured by the CAR-T Nanostring panel. Data shows Nanostring normalised counts for the top 20 HIF1A responsive genes reported by Smelc *et al*[27] (left plot) and by Oki *et al*[26] (right plot). Data from donor matched *ex vivo* mTregs, expTreg^{x2} and expTreg$^{x5\text{-}6}$ from 3 donors and expTreg^{x2} (expTreg^{x2restim}) from 2 donors are shown.

b-c: The expression of 45 TCR alpha and 46 TCR beta variable chains was measured in *ex vivo* mTregs, expTreg^{x2} and expTreg$^{x5\text{-}6}$ from 3 donors. Log2 normalised counts measured by Nanostring are displayed and the green dotted line represents the mean of the *ex vivo* dataset.

a – example gating of expTregs using an FMO control to set CD73 threshold

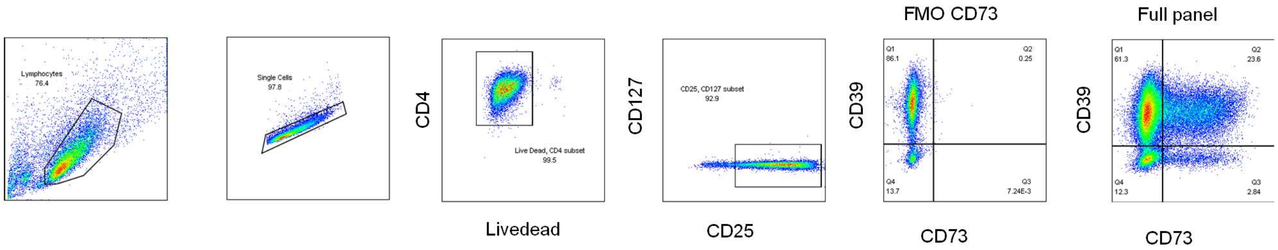

b - gain of CD73 by expTregs over time

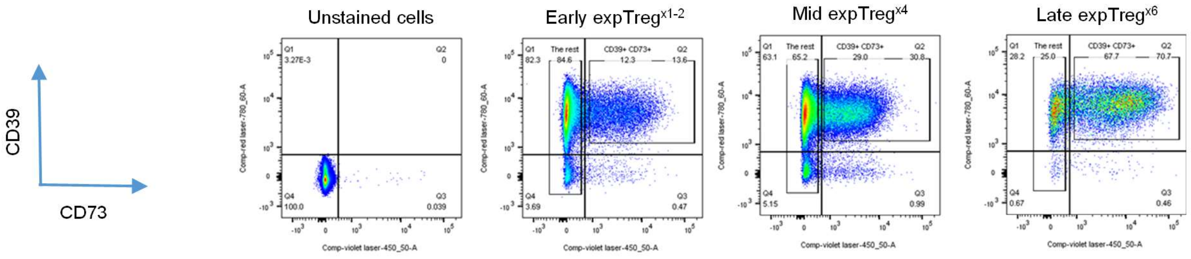

c – example FMO thresholding for FOXP3 expression in expTregs and FoxP3 stability over time

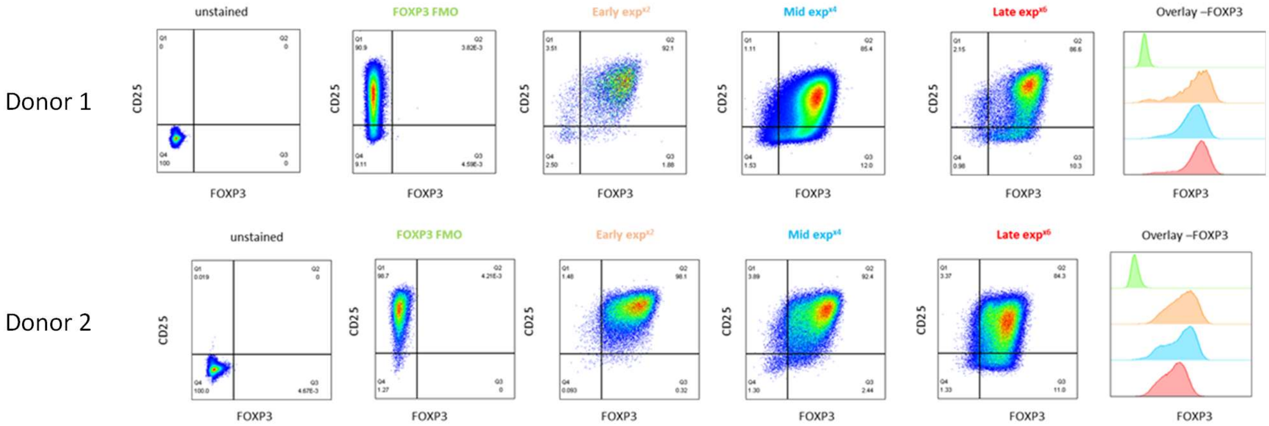

Supplementary Figure 8:

Control staining of CD73 and FOXP3 over time and thresholding using FMOs

a: Example gating of expTregs using a fluorescence minus one (FMO) control to set CD73 threshold.

b: Representative example of gain of CD73 over time in expTregs compared to unstained control cells.

c: Example thresholding for FoxP3 expression based on unstained cells and fluorescence minus one control (FMO) for FoxP3 in 2 donors, and stability of FoxP3 over time in early (^{x2}), mid (^{x4}) and late (^{x6}) expanded Tregs. Histograms show comparative FoxP3 MFI over time.
