# Supplementary material for: Therapeutically expanded human regulatory T-cells are super-suppressive due to HIF1A induced expression of CD73
Source: Commun Biol. 2021 Oct 14;4:1186. doi: 10.1038/s42003-021-02721-x (PMC8516976; doi:10.1038/s42003-021-02721-x)
Supplement: Supplementary file 2 — Description of Additional Supplementary Files [file 42003_2021_2721_MOESM2_ESM.pdf]

Description of Additional Supplementary Files

File name: Supplementary Data 1.
Description: Raw and normalised gene expression counts measured by Nanostring for the ex vivo mTregs, expTregsx2, expTregsx5 and expTregsx2restim as well as the flow-sorted CD39+CD73+ DP and non-DP expTregs. Differential expression between each Treg condition is shown on a separate sheet.

File name: Supplementary Data 2.
Description: Gene Set Enrichment Analysis of Nanostring data, performed using the GSEA tool, and enrichment in the genesets from Hallmark, Reactome and GO Biological Processes.

File name: Supplementary Data 3.
Description: Source data used to create manuscript figures.
