# Supplementary material for: Therapeutically expanded human regulatory T-cells are super-suppressive due to HIF1A induced expression of CD73
Source: Commun Biol. 2021 Oct 14;4:1186. doi: 10.1038/s42003-021-02721-x (PMC8516976; doi:10.1038/s42003-021-02721-x)
Supplement: Supplementary file 6 — Reporting Summary [file 42003_2021_2721_MOESM6_ESM.pdf]

nature research

Corresponding author(s): Dr Joanne Jones
Department of Clinical Neurosciences
Box 165, Clifford Allbutt Building
Cambridge Biomedical Campus
University of Cambridge, UK
jls53@medschl.cam.ac.uk

Last updated by author(s): Sept 10th 2021

Reporting Summary

Nature Research wishes to improve the reproducibility of the work that we publish. This form provides structure for consistency and transparency in reporting. For further information on Nature Research policies, see our Editorial Policies and the Editorial Policy Checklist.

Statistics

For all statistical analyses, confirm that the following items are present in the figure legend, table legend, main text, or Methods section.

n/a	Confirmed	
☐	☒	The exact sample size (*n*) for each experimental group/condition, given as a discrete number and unit of measurement
☐	☒	A statement on whether measurements were taken from distinct samples or whether the same sample was measured repeatedly
☐	☒	The statistical test(s) used AND whether they are one- or two-sided<br>*Only common tests should be described solely by name; describe more complex techniques in the Methods section.*
☐	☒	A description of all covariates tested
☐	☒	A description of any assumptions or corrections, such as tests of normality and adjustment for multiple comparisons
☐	☒	A full description of the statistical parameters including central tendency (e.g. means) or other basic estimates (e.g. regression coefficient) AND variation (e.g. standard deviation) or associated estimates of uncertainty (e.g. confidence intervals)
☐	☒	For null hypothesis testing, the test statistic (e.g. *F*, *t*, *r*) with confidence intervals, effect sizes, degrees of freedom and *P* value noted<br>*Give P values as exact values whenever suitable.*
☐	☒	For Bayesian analysis, information on the choice of priors and Markov chain Monte Carlo settings
☐	☒	For hierarchical and complex designs, identification of the appropriate level for tests and full reporting of outcomes
☐	☒	Estimates of effect sizes (e.g. Cohen's *d*, Pearson's *r*), indicating how they were calculated

Our web collection on statistics for biologists contains articles on many of the points above.

Software and code

Policy information about availability of computer code

Data collection	Flow cytometry data were acquired on a BD LSR Fortessa or CantoII with BD FACSDIVA software (v8). Mass Spectrometry data was analyzed using a Thermo Quantiva interfaced with a Ultra High performance Liquid Chromatography (UHPLC) Vanquish system (Thermo Scientific, Hemel Hempstead, UK). Metabolism assays were performed on a 96-well XF extracellular flux analyzer (EFA) (Seahorse Bioscience). Lactate was measured using a Dimension EXL autoanalyser (Siemens, Germany) with Flex© reagent cartridges (product code DF16). Nanostring data was acquired using an nCounter prep station and digital analyser (Nanostring Technologies).
Data analysis	Flow cytometric data was analyzed using Flowjo software (v7.6.5-v10.5.3)<br>Statistical analyses were performed using Graphpad Prism (v6) or Matlab 2017a, (The Mathworks, MA)<br>Nanostring data were analyzed using nsolver 4.0 software (Nanostring), EnhancedVolcano R package Blighe, K. kevinblighe/EnhancedVolcano. (2020)., Bioinfokit python package Renesh Bedre. reneshbedre/bioinfokit: Bioinformatics data analysis and visualization toolkit. (Zenodo, 2020). doi:10.5281/zenodo.3841708.

For manuscripts utilizing custom algorithms or software that are central to the research but not yet described in published literature, software must be made available to editors and reviewers. We strongly encourage code deposition in a community repository (e.g. GitHub). See the Nature Research guidelines for submitting code & software for further information.

Data

Policy information about availability of data

All manuscripts must include a data availability statement. This statement should provide the following information, where applicable:

- Accession codes, unique identifiers, or web links for publicly available datasets
- A list of figures that have associated raw data
- A description of any restrictions on data availability

All raw nanostring data are available in supplementary information. All other data are available from the corresponding author upon reasonable request.

Field-specific reporting

Please select the one below that is the best fit for your research. If you are not sure, read the appropriate sections before making your selection.

☒ Life sciences ☐ Behavioural & social sciences ☐ Ecological, evolutionary & environmental sciences

For a reference copy of the document with all sections, see nature.com/documents/nr-reporting-summary-flat.pdf

Life sciences study design

All studies must disclose on these points even when the disclosure is negative.

Sample size	No statistical analyses were performed to determine sample size. Sample sizes were determined based on our previous experience, consistency of the data and the literature in this field.
Data exclusions	No data was excluded
Replication	All technical repeats were included in analysis, all data was replicated and no data excluded
Randomization	No randomization has been done as all experiments involved a side-by-side comparison of ex vivo vs expTregs in all conditions tested.
Blinding	Blinding was not relevant since analyses of the controls and the experimental conditions were performed using the same gating strategies. In addition, cells from every donor underwent the same experimental procedure in a side-by-side comparison.

Reporting for specific materials, systems and methods

We require information from authors about some types of materials, experimental systems and methods used in many studies. Here, indicate whether each material, system or method listed is relevant to your study. If you are not sure if a list item applies to your research, read the appropriate section before selecting a response.

Materials & experimental systems

n/a	Involved in the study	
☐	☒	Antibodies
☐	☐	Eukaryotic cell lines
☐	☐	Palaeontology and archaeology
☐	☒	Animals and other organisms
☐	☒	Human research participants
☐	☐	Clinical data
☐	☐	Dual use research of concern

Methods

n/a	Involved in the study	
☐	☐	ChIP-seq
☐	☒	Flow cytometry
☐	☐	MRI-based neuroimaging

Antibodies

Antibodies used

Antibodies uses in this study are described fully in the methods section:
CD39-BB515 (clone Tu66, BD) 1:50 dilution
CD39-FITC (clone ebioA1, ebioscience/thermofisher 11-0399-42) 1:50 dilution
CD25-PE (clone 2A3, BD) 1:50 dilution
CD25-PE (clone MA251, BD) 1:10 dilution
CD25-APC (clone 2A3, BD) 1:50 dilution
CD25-APC (clone MA251, BD) 1:10 dilution
CD4 APCFire/750 (Clone A161A1, Biolegend) 1:50 dilution
CD4-ECD (Clone SFCI12T4D11, Beckman Coulter)
CD73-BV421 (clone AD2, Biolegend) 1:50 dilution
CD8 V500 (clone RPA-T8, BD BD560774) 1:50 dilution
CD3-BV605 (SK7, Biolegend) 1:50 dilution

	CD127-AF647 (clone HIL-7R-M21, BD) 1:50 dilution<br>CD127-PEcy7 (eBioRDR5, ebioscience/Thermofisher) 1:50 dilution<br>CD45RA-BV786 (clone Hi100, BD) 1:50 dilution<br>CTLA-4 BV421 (clone, BN13, Biolegend BL369606) 1:50 dilution<br>CD3 BV570 (clone UCHT1, Biolegend BL300436) 1:50 dilution<br>CD45RA BV605 (clone HI100, BL304134) 1:50 dilution<br>CD39 BV650 (clone Tu66, BD BD563681) 1:50 dilution<br>CD73 BV786 (clone AD2, BD BD344028) 1:50 dilution<br>TIGIT PerCP-efl710 (clone MBSA43, ebio 46-9500-42) 1:50 dilution<br>CD127 PE-Cy7 (clone eBioRDR5, eBio 25-1278-42) 1:50 dilution<br>CD4 APC-R700 (clone RPA-T4, BD BD564976) 1:50 dilution<br>CD226 APC-Fire750 (clone 11A8, Biolegend BL338320) 1:50 dilution<br>HELIOS FITC (clone 22F6, Biolegend BL137214) 1:50 dilution<br>CTLA4 PE-Dazzle (clone BN13, Biolegend BL369616) 1:50 dilution<br>FOXP3-PE (clone PCH1101 eBio 12-4776-41) 1:50 dilution<br>FOXP3-PE (clone 259D Biolegend BL320207) 1:50 dilution<br>Ki67 BUV395 (clone B56, BD BD564071) 1:50 dilution<br>CD70 BV786 (clone Ki-24, BD 565338) 1:50 dilution<br>CD27 BUV395 (clone L128 BD 563815) 1:50 dilution<br>CD39 APCcy7 (clone A1, BL 328225) 1:50 dilution
Validation	All antibodies used in this manuscript were obtained from commercial providers and have been validated by the manufacturer for intended application as well as being used on other peer-reviewed studies. In addition, all antibodies used for this study had been tested against suitable positive and negative control cell populations to validate them prior to use.

Eukaryotic cell lines

Policy information about cell lines

Cell line source(s)	*State the source of each cell line used.*
Authentication	*Describe the authentication procedures for each cell line used OR declare that none of the cell lines used were authenticated.*
Mycoplasma contamination	*Confirm that all cell lines tested negative for mycoplasma contamination OR describe the results of the testing for mycoplasma contamination OR declare that the cell lines were not tested for mycoplasma contamination.*
Commonly misidentified lines<br>(See ICLAC register)	*Name any commonly misidentified cell lines used in the study and provide a rationale for their use.*

Palaeontology and Archaeology

Specimen provenance	*Provide provenance information for specimens and describe permits that were obtained for the work (including the name of the issuing authority, the date of issue, and any identifying information).*
Specimen deposition	*Indicate where the specimens have been deposited to permit free access by other researchers.*
Dating methods	*If new dates are provided, describe how they were obtained (e.g. collection, storage, sample pretreatment and measurement), where they were obtained (i.e. lab name), the calibration program and the protocol for quality assurance OR state that no new dates are provided.*

☐ Tick this box to confirm that the raw and calibrated dates are available in the paper or in Supplementary Information.

Ethics oversight	*Identify the organization(s) that approved or provided guidance on the study protocol, OR state that no ethical approval or guidance was required and explain why not.*

Note that full information on the approval of the study protocol must also be provided in the manuscript.

Animals and other organisms

Policy information about studies involving animals; ARRIVE guidelines recommended for reporting animal research

Laboratory animals	C57BL/6 mice (males and females) and BALB/c Rag2-/- IL2rg-/- aged 6-12 weeks old were used in this study.
Wild animals	None
Field-collected samples	None
Ethics oversight	All mice were treated in strict accordance to the UK Animals (Scientific Procedures) Act of 1986 and under PPLs P8869535A or PPL80/8970

Note that full information on the approval of the study protocol must also be provided in the manuscript.

Human research participants

Policy information about studies involving human research participants

Population characteristics	Healthy adults aged above 18 years and both males and females were used as donors in this study. A subgroup of participants were genotyped for the ENTDP1 SNP rs10748643
Recruitment	Participants were recruited in keeping with ethically approved study protocols. Blood samples were obtained from the NHS blood & transfusion service where consent was taken at the time of blood donation or from healthy controls working within the University of Cambridge. Human spleen samples were obtained from deceased organ donors via the Cambridge Biorepository for Translational Medicine (CBTM). Healthy skin and blood was also donated from patients undergoing plastic surgery procedures in Oxford. Written consent was obtained for all participants.
Ethics oversight	11/EE/0007 REC: East of England-Central Cambridge Research Committee; 07/H0605/130: Oxfordshire Research Ethics Committee and 15/EE/0152 REC: East of England-Central Cambridge Research Committee

Note that full information on the approval of the study protocol must also be provided in the manuscript.

Clinical data

Policy information about clinical studies

All manuscripts should comply with the ICMJE guidelines for publication of clinical research and a completed CONSORT checklist must be included with all submissions.

Clinical trial registration	*Provide the trial registration number from ClinicalTrials.gov or an equivalent agency.*
Study protocol	*Note where the full trial protocol can be accessed OR if not available, explain why.*
Data collection	*Describe the settings and locales of data collection, noting the time periods of recruitment and data collection.*
Outcomes	*Describe how you pre-defined primary and secondary outcome measures and how you assessed these measures.*

Dual use research of concern

Policy information about dual use research of concern

Hazards

Could the accidental, deliberate or reckless misuse of agents or technologies generated in the work, or the application of information presented in the manuscript, pose a threat to:

No	Yes	
☒	☐	Public health
☒	☐	National security
☒	☐	Crops and/or livestock
☒	☐	Ecosystems
☒	☐	Any other significant area

Experiments of concern

Does the work involve any of these experiments of concern:

No	Yes	
☒	☐	Demonstrate how to render a vaccine ineffective
☒	☐	Confer resistance to therapeutically useful antibiotics or antiviral agents
☒	☐	Enhance the virulence of a pathogen or render a nonpathogen virulent
☒	☐	Increase transmissibility of a pathogen
☒	☐	Alter the host range of a pathogen
☒	☐	Enable evasion of diagnostic/detection modalities
☒	☐	Enable the weaponization of a biological agent or toxin
☒	☐	Any other potentially harmful combination of experiments and agents

ChIP-seq

Data deposition

☐ Confirm that both raw and final processed data have been deposited in a public database such as GEO.

☐ Confirm that you have deposited or provided access to graph files (e.g. BED files) for the called peaks.

Data access links<br>*May remain private before publication.*	*For "Initial submission" or "Revised version" documents, provide reviewer access links. For your "Final submission" document, provide a link to the deposited data.*
Files in database submission	*Provide a list of all files available in the database submission.*
Genome browser session<br>(e.g. UCSC)	*Provide a link to an anonymized genome browser session for "Initial submission" and "Revised version" documents only, to enable peer review. Write "no longer applicable" for "Final submission" documents.*

Methodology

Replicates	*Describe the experimental replicates, specifying number, type and replicate agreement.*
Sequencing depth	*Describe the sequencing depth for each experiment, providing the total number of reads, uniquely mapped reads, length of reads and whether they were paired- or single-end.*
Antibodies	*Describe the antibodies used for the ChIP-seq experiments; as applicable, provide supplier name, catalog number, clone name, and lot number.*
Peak calling parameters	*Specify the command line program and parameters used for read mapping and peak calling, including the ChIP, control and index files used.*
Data quality	*Describe the methods used to ensure data quality in full detail, including how many peaks are at FDR 5% and above 5-fold enrichment.*
Software	*Describe the software used to collect and analyze the ChIP-seq data. For custom code that has been deposited into a community repository, provide accession details.*

Flow Cytometry

Plots

Confirm that:

☒ The axis labels state the marker and fluorochrome used (e.g. CD4-FITC).

☒ The axis scales are clearly visible. Include numbers along axes only for bottom left plot of group (a 'group' is an analysis of identical markers).

☒ All plots are contour plots with outliers or pseudocolor plots.

☒ A numerical value for number of cells or percentage (with statistics) is provided.

Methodology

Sample preparation	Cells analyzed by flow cytometry were either Peripheral blood or spleen mononuclear cells (PBMC/SMNC) isolated from human or murine blood/spleen by ficoll density gradient centrifugation or ex vivo Tregs sorted by FACS as described in the methods or Tregs expanded in vitro using standard Treg expansion protocols. Cells were washed with PBS/1%FCS FACS buffer and stained for 20 minutes on ice for surface markers followed (where required) by permeablization and fixation using the Invitrogen FOXP3 intracellular staining kit and stained for intracellular markers according to manufacturer's instructions and as detailed in the methods section. Samples were washed into PBS/1%FCS for analysis/acquisition.
Instrument	BD LSR Fortessa or Canto II running FACSDIVA software
Software	FACSDiva for acquisition and Flowjo V.10.5.3 for analysis
Cell population abundance	Abundance of the ex vivo sorted Tregs in PBMC was approximately 5% and the purity of the post sorted fractions were > 95% as detailed in both sort reports and by subsequent Treg phenotyping of the cultured sorted Tregs at regular intervals during their post-sort expansion.
Gating strategy	Gating strategies are detailed in the supplementay information. Briefly, the primary gating for Tregs was: lymphocytes>single cells>live TCD3+ cells>CD4+CD25HiCD127low Tregs. For suppression assays Teffectors and Tregs were distinguished on the basis of separate cell proliferation dye fluorchormes (V670- Tregs; V450 - Teffs) and Live Non Tregs V670- populations analysed for proliferation. Unstained cells and/or FMOs were used to set negative gates/quadrants.

☒ Tick this box to confirm that a figure exemplifying the gating strategy is provided in the Supplementary Information.

Magnetic resonance imaging

Experimental design

Design type	*Indicate task or resting state; event-related or block design.*
Design specifications	*Specify the number of blocks, trials or experimental units per session and/or subject, and specify the length of each trial or block (if trials are blocked) and interval between trials.*
Behavioral performance measures	*State number and/or type of variables recorded (e.g. correct button press, response time) and what statistics were used to establish that the subjects were performing the task as expected (e.g. mean, range, and/or standard deviation across subjects).*

Acquisition

Imaging type(s)	*Specify: functional, structural, diffusion, perfusion.*
Field strength	*Specify in Tesla*
Sequence & imaging parameters	*Specify the pulse sequence type (gradient echo, spin echo, etc.), imaging type (EPI, spiral, etc.), field of view, matrix size, slice thickness, orientation and TE/TR/flip angle.*
Area of acquisition	*State whether a whole brain scan was used OR define the area of acquisition, describing how the region was determined.*

Diffusion MRI ☐ Used ☐ Not used

Preprocessing

Preprocessing software	*Provide detail on software version and revision number and on specific parameters (model/functions, brain extraction, segmentation, smoothing kernel size, etc.).*
Normalization	*If data were normalized/standardized, describe the approach(es): specify linear or non-linear and define image types used for transformation OR indicate that data were not normalized and explain rationale for lack of normalization.*
Normalization template	*Describe the template used for normalization/transformation, specifying subject space or group standardized space (e.g. original Talairach, MNI305, ICBM152) OR indicate that the data were not normalized.*
Noise and artifact removal	*Describe your procedure(s) for artifact and structured noise removal, specifying motion parameters, tissue signals and physiological signals (heart rate, respiration).*
Volume censoring	*Define your software and/or method and criteria for volume censoring, and state the extent of such censoring.*

Statistical modeling & inference

Model type and settings	*Specify type (mass univariate, multivariate, RSA, predictive, etc.) and describe essential details of the model at the first and second levels (e.g. fixed, random or mixed effects; drift or auto-correlation).*
Effect(s) tested	*Define precise effect in terms of the task or stimulus conditions instead of psychological concepts and indicate whether ANOVA or factorial designs were used.*

Specify type of analysis: ☐ Whole brain ☐ ROI-based ☐ Both

Statistic type for inference (See Eklund et al. 2016)	*Specify voxel-wise or cluster-wise and report all relevant parameters for cluster-wise methods.*
Correction	*Describe the type of correction and how it is obtained for multiple comparisons (e.g. FWE, FDR, permutation or Monte Carlo).*

Models & analysis

n/a	Involved in the study
☐	☐ Functional and/or effective connectivity
☐	☐ Graph analysis
☐	☐ Multivariate modeling or predictive analysis

Functional and/or effective connectivity	*Report the measures of dependence used and the model details (e.g. Pearson correlation, partial correlation, mutual information).*
Graph analysis	*Report the dependent variable and connectivity measure, specifying weighted graph or binarized graph, subject- or group-level, and the global and/or node summaries used (e.g. clustering coefficient, efficiency, etc.).*

Multivariate modeling and predictive analysis

Specify independent variables, features extraction and dimension reduction, model, training and evaluation metrics.
